# Context Size and Set Size Effects: The Relevance of Specific Cues When Searching Long-Term Memory

**DOI:** 10.1007/s42113-025-00255-7

**Published:** 2025-08-07

**Authors:** Susanne Haridi, Eric Schulz, Mirko Thalmann

**Affiliations:** 1 Institute for Human-Centered AI, Helmholtz Munich, Ingolstädter Landstraße 1, D-85764 Neuherberg, Germany; 2 Max Planck School of Cognition, Stephanstr. 1A, D-04103 Leipzig, Germany

**Keywords:** Search, Long-term memory, Retrieval, Recall, Similarity, Set size, Complexity

## Abstract

Retrieving the relevant information from our knowledge and experiences poses a challenging problem to our memory system. In this study, we explore how retrieval of specific pieces of information from long-term memory is affected by the number of items to be remembered and by the characteristics of retrieval cues. Specifically, we looked at whether an increased semantic similarity between cue and target improves recall and whether additional context cues aid retrieval depending on their features and specificity. Using a cued-recall task, we observed across three experiments that increasing the number of learned word pairs slowed reaction times (RTs) and reduced retrieval accuracy. This effect was modulated by context: set size effects did not extend across multiple, unrelated lists. Semantic similarity between cues and targets consistently facilitated retrieval, independent of set size. Experiment 2 revealed no effect of additional visual context cues on RTs, while Experiment 3 demonstrated that larger semantic contexts hindered recall, leading to slower RTs and lower accuracy compared to unrelated lists. These findings are consistent with predictions from a model of memory portraying retrieval as a sequential search through a semantic network.

## Introduction

What is your mother’s name? Who is the current president of the USA? How much do you pay for your monthly cell phone contract? Chances are, you recalled the answers to these questions before you even finished reading. But how does this happen so effortlessly? If long-term memory (LTM) was merely a box, in which all memories were thrown together without any order, locating the right piece of information would become a daunting and time-consuming process, especially as more and more memories accumulate over time. This clear mismatch between a disorganized model of memory and our ability to quickly retrieve relevant information suggests that LTM must be structured in a way that allows for efficient access. A refined question, then, is as follows: how is memory organized in a way that supports such rapid retrieval? This is particularly relevant since as we grow older the number of memories we store only grows larger. In this study, we use retrieval times in a cued-recall paradigm to investigate (a) how the retrieval of specific memories from LTM is affected by the number of memory items to be remembered, (b) whether additional context cues help us retrieve specific items faster, and (c) whether successful retrieval depends on the specificity of these cues. We discuss the changes in retrieval times tell us about the mechanisms of the search and the organization of LTM. We use a cued-recall task to do this, because it is a task, in which we have to search for a specific piece of information in memory. In doing so, we extend previous work by shifting the focus from response accuracies to retrieval times.

### Set Size Effects

We are not the first to consider that the amount of information stored in memory could negatively influence retrieval processes. Set size effects (aka list-length effects) have long been shown in experiments varying the number of items to be learned. Here, set size effects have been demonstrated in all three typically studied LTM tasks: (1) recognition tasks, in which participants have to distinguish between previously presented and new items (Ratcliff & Murdock, 1976; Strong Jr, 1912; 2) free recall tasks, in which all presented items need to be recalled in any order (Gillund & Shiffrin, 1981; Grenfell-Essam et al., 2013; Mack et al., 2017; Ward, 2002; Ward et al., 2010); and (3) cued-recall tasks, in which an item corresponding to a particular cue needs to be recalled (Tulving & Pearlstone, 1966). However, with few exceptions (Diller et al., 2001; Nobel & Shiffrin, 2001), this effect has rarely been studied in RTs, which are particularly helpful in distinguishing between a parallel and a serial search mechanism, which is not possible with accuracy. Importantly, there are also results that indicate that a set size effect might not exist. Buratto and Lamberts (2008) for example did not find an effect of set size for recognition paradigms. Dennis and Humphreys (2001) and Kinnell and Dennis (2011) even argued and showed that set size effects in recognition memory disappear in if you control for certain confounds such as the retention interval, attention, displaced rehearsal, and contextual reinstatement. Accordingly, it is not clear if set size effects exist, particularly for the less studied RTs.

The idea that the volume of stored information can impair retrieval has also been proposed as a mechanism underlying age-related memory decline. Ramscar et al. (2014) made a strong argument that a decline of performance in psychometric tests observed in older adults can be well-explained by the increasing search demands that come with more experiences. This notion is consistent with the copious evidence showing that with age memory performance declines (Old & Naveh-Benjamin, 2008; Zacks et al., 2000).

However, memories relying on well-practiced experiences are less affected by age than memories that require new connections (Burke & MacKay, 1997). Additionally, age deficits in associative memory are reduced if there is an existing semantic relationship between the associated items (Naveh-Benjamin et al., 2003). This suggests that not only the amount of memories, but also how well they can be integrated into an existing network matters. In other words, memory problems due to a growing number of memories can potentially be overcome by the semantic organization of memory.

### The Organization of Memory

It has been proposed that memory is a high-dimensional semantic space (for a recent review see Kumar, 2021).

Building on the idea of a semantic organization, Shiffrin and Atkinson (1969) proposed that each stored memory is “self-addressable,” meaning that the location in which a memory is stored depends on its content. In other words, each memory item is given a meaningful location in this memory space. In such a system, the content of a cue provides likely locations in which to look for corresponding memories. In the field of memory research, it has long been assumed that restricting memory retrieval to relevant information can be achieved by focusing on memories that are in some way similar to the cue (Shiffrin & Steyvers, 1997). In other words, knowing where you encode a memory in a semantic network also means you know where to find it and related memories. If true, to ascertain if we have seen a given object before we would just have to look in the place in memory, where we would encode that object. If something is already there, we know we have seen it before, enabling fast recognition. When recalling information from LTM, the memory cue and the target memory are not the same. In the proposed memory system, similarity to a memory cue would determine which items are considered.

This idea is consistent with models such as the context maintenance and retrieval model for free recall (TMR; Polyn et al., 2009) which builds on the temporal context model (TCM; Howard & Kahana, 2002a). These models propose that memory retrieval does not operate across the entire set of stored memories, but is instead driven by internally maintained context representations, which guide the search to related items. This supports the notion that it is the local competition, meaning the number of memories similar to or associated with the cue, that limits performance. In line with cue overload theory (Watkins & Watkins, 1975), we argue that recall becomes harder as the specificity of the cue declines.

Accordingly, the usefulness of a particular cue can reveal a lot about how memory is organized.

#### Semantic Similarity as an Organizational Principle of LTM

But what makes memories similar? Previous research has identified a few key dimensions, which are relevant for the organization of memory: The distance between representations in a semantic network (e.g., Hills et al., 2012; Howard & Kahana, 2002b), the time since the information has been encountered (e.g. Dosher, 1981), and the context in which the information was initially learned (e.g. Godden & Baddeley, 1975). In what follows, we review the work most relevant to the current investigation regarding all three dimensions.

Memory research has long highlighted how semantics are important when retrieving information from LTM. In free recall tasks, additional category labels (Tulving & Pearlstone, 1966) and additional context or category names (Hudson & Austin, 1970) improve recall accuracy. In cued-recall tasks, focusing on the sematic relationship between cue and target (Epstein et al., 1975) and even just increased processing through judgments of learning (Janes et al., 2018) result in better recall. In addition to the semantic similarity, Cornell et al. (2024) showed that the usefulness of a cue depends on the semantic and temporal similarity of a cue word to the remaining target words, highlighting that similarity in memory is multi-dimensional.

The idea that semantic categories help retrieve information from LTM is also supported by findings from fluency tasks, in which participants are asked to name as many members from a category (e.g., animals) as possible in a limited amount of time. The findings suggest that people continuously recall words from one semantic category before moving on to a different category, suggesting that recalling one item activates related items within the same semantic cluster (Hills et al., 2012; Troyer et al., 1997). This behavior aligns with models of memory that assume that semantic proximity facilitates access, such that items that are semantically or associatively close are more likely to be retrieved in succession (Collins & Loftus, 1975; Howard & Kahana, 2002a).

However, while semantic similarity can aid recall, it may also hinder it through interference when many items are associated with the same cue. Some studies find that larger semantic categories reduce recall accuracy in free recall (Patterson, 1972) and recognition (Shiffrin et al., 1995), and that lists composed of related words suffer from output interference and retrieval competition (Roediger & Schmidt, 1980; Anderson & Neely, 1996; Anderson, 1974). According to the encoding specificity principle (Tulving & Thomson, 1973) and Tulving’s theory of cue-dependent retrieval (Tulving & Thomson, 1973; Tulving & Pearlstone, 1966), this is because only cues that closely match the encoding context are effective. Therefore, when a cue is overloaded with associations, retrieval suffers (Watkins & Watkins, 1975). These findings underscore that retrieval is most efficient when semantic cues are specific and diagnostic of the target memory.

This extensive research on the role of semantic similarity has led to a good understanding of many of the principles that govern memory retrieval. In summary, we know that the more items are associated with a cue, the harder it is to retrieve any one of them (Tulving & Pearlstone, 1966; Watkins & Watkins, 1975), that a cue is only useful if it matches how the memory was encoded, that semantic proximity helps recall, and that competition among similar memories can hurt performance. However, we still do not fully understand how the cue’s similarity to both target and non-target items affects recall accuracy, and in particular recall speed.

Accordingly, it is important to further investigate potential dimensions of similarity.

#### (Temporal) Context as an Organizational Principle of LTM

As famously illustrated by Godden and Baddeley’s diver study (Godden & Baddeley, 1975), context seems to play an important role in our ability to recall information from memory (Heald et al., 2023). While individual studies may vary in their findings, meta-analytic evidence highlights the consistent influence of context across diverse memory paradigms (Smith & Vela, 2001). However, defining context is difficult. A general definition might state that context is the circumstances that form the setting for an event, statement, or idea, and in terms of which it can be fully understood. Such a definition would imply that the context is semantically related to the memory. In psychology experiments, however, context is often operationalized by the presence of non-task related additional features. As such, a context might be a background picture, an auditory cue, a virtual reality environment (Pacheco et al., 2017; Shin et al., 2021), a video (Steven et al., 2014), or a physical location change like in the diver study (Godden & Baddeley, 1975).

But contexts too are not always very specific in what memories they cue to. A context feature that changes slowly over time or has a semantic meaning might be associated with many memory items. In such cases, providing additional context cues might be detrimental.

Furthermore, different context manipulations seem to have varying effects on their relevance for retrieval (Wickens, 1970). In fact, some researchers have argued against a general effect of context, stating that the helpfulness of context might be strongly modulated by what is perceived as critical context features from an individual’s point of view (Fernandez & Glenberg, 1985). But are there context features relevant for everyone? Among the many potential features, time stands out as a universally relevant factor (Brown et al., 2007). We naturally tend to remember information encountered more recently. There is also evidence that behaviorally relevant or semantically related context features are particularly helpful in aiding recall (Cornell et al., 2024). But non-task related features, like additional background pictures might also aid memory in some cases (Smith & Vela, 2001; Liu et al., 2024).

Regardless of whether the context is semantically meaningful or not, it can be viewed as additional cues that increase or decrease the overall similarity between a memory cue and a memory target. As such, distinguishing between cue and context is somewhat arbitrary, but it can be helpful when trying to delineate between what aspects of a cue facilitate retrieval. Accordingly, when we refer to additional (context) cues, what we are really referring to is the aspects of the context, which we experimentally manipulated. In other words, we view context as an umbrella term that includes a diverse set of features. As such, it is not surprising that people find varying effects of context given its imprecise definition. Instead, we argue for the need to explicitly examine and describe which features of a larger context are relevant for successful memory retrieval. Such features could be semantic relatedness, external perceptibility (seen, felt, heard, tasted or smelled context featured vs., e.g., internal state such as the current mood) or task-relevancy. Based on the existing theories we outlined above, we propose that the usefulness of the additional cues a context provides depends on two factors: (1) How much does the context increase the similarity between cue and target? (2) How distinctly is the context associated with the target?

In this study, we investigate whether the helpfulness of additional context features depends on the size of the context, or in other words, whether the specificity of the context matters for memory performance. As mentioned, this effect has already been described in the literature. However, here, we are interested in whether set size effects are just due an increased similarity which is based on the existence of a context cues and whether these set size effects depend on the features of the context. In doing so, we want to explicitly address the question of whether potential set size effects arise based on the number of items which are similar to the cue (which includes the context).

To do so, we investigate the effect of three context features:

1. Temporal list context: This context is task-relevant, as participants only ever need to recall items from the most recent list, and is therefore likely to be used. Moreover, prior research suggests that temporal context elicits robust effects, which could potentially reduce interference from precious experiences and result in list specific set size effects.
2. Visual context: This manipulation is designed to be incidental and task-irrelevant. Here, participants are exposed to distinct visual backgrounds during learning and recall, which are not semantically or structurally meaningful for the task. This allows us to test whether the mere presence of perceptual context alone can modulate recall, especially in the absence of explicit task relevance. If visual context produces context specific set size effects, it would suggest that even non-diagnostic, passive cues can serve as retrieval scaffolds, possibly through reinstatement mechanisms or cue-dependent attention shifts.
3. Semantic context: Based on previous research, items from the same semantic categories are likely organized together in memory. However, unlike temporal context, semantic associations can persist over experiences, making them a pervasive and flexible source of retrieval structure. If semantic context produces strong context specific set size effects, it would suggest that memory search is sensitive to the meaningful structure of information, not just its temporal proximity. Furthermore, because semantic similarity can increase competition among related items, this manipulation allows us to explore how fine-grained semantic cues either facilitate or hinder recall depending on how densely populated the context is.

Together, these three manipulations allow us to compare contexts that vary in both strength and task relevance. This helps us determine not only whether context matters, but which kinds of context are most influential in shaping set size effects and why.

### Episodic and Semantic Memory

Before summarizing the aim of our study, we briefly address the conventional distinction between semantic and episodic memory. Semantic memory is typically defined as general knowledge about the world, devoid of contextual information (e.g., knowing that “dog” translates to “Hund” in German), while episodic memory refers to the recollection of specific events situated in time and space (e.g., remembering the moment you learned that fact; Tulving et al., 1972; Tulving, 1983).

While this might be intuitive, this distinction can become murky in many situations. For example, if you just learned the word “Hund,” your ability to recall it may involve semantic retrieval, episodic recall, or a blend of both. Another such example is cherished memory. If you recalled an episode often enough for it to become knowledge, e.g., the time your grandma gave you a yellow plush dog, then is this still episodic memory (the recall of the event) or is it rather a semantic memory (the knowledge that your grandma gave you a yellow plush dog).

Rather than this binary distinction, in many cases, it may be more productive to view episodic and semantic memory as interrelated processes, differing not in kind but in degree and function (e.g., Greenberg & Verfaellie, 2010). Our study also avoids a direct classification and instead emphasizes how the type of retrieval cues shape what memories are recalled.

### Summary

In summary, the notion that memory retrieval is cue-dependent and shaped by the similarity structure of memory has a long history in cognitive psychology (Tulving & Pearlstone, 1966; Tulving & Thomson, 1973; Watkins & Watkins, 1975). Similarly, much research has been done on the organizational structure of memory, showing that semantic similarity and temporal context can shape memory retrieval (Roediger & Schmidt, 1980; Howard & Kahana, 2002a).

Building on this well-established foundation, we want to revisit these classic questions with a focus on RTs, which, to our knowledge, have only been analyzed in a small number of studies (e.g. Nobel & Shiffrin, 2001; Diller et al., 2001) to investigate set size effects in cued recall. We believe RTs are a helpful addition to accuracy measures (Ratcliff, 1978) when distinguishing between memory theories.

In this study, we ask the following: (1) How are reaction times in cued recall from long-term memory affected by the number of stored memory items? And (2) what features modulate the time required to retrieve a specific memory item? Specifically, we examine (a) whether increased semantic similarity between cue and target speeds up retrieval, (b) whether temporal context functions as an organizing principle that may limit the effective search space and reduce set size effects, and (c) whether additional retrieval cues facilitate retrieval and to what extent this benefit depends on the cue’s specificity and the size of the associated category.

Although the effects of cue specificity and category size on recall are well-documented (e.g., Tulving & Pearlstone, 1966; Patterson, 1972), our contribution lies in providing new reaction time evidence with added controls for confounds (Kinnell & Dennis, 2011), which was not done in previous studies using cued recall, and comparisons to specifically designed control conditions that help to clarify how and when additional cues help or hinder memory search. In doing so, we aim to better understand the mechanisms that support the efficient and selective retrieval we routinely observe in everyday memory use.

## A Sequential Model of Memory Retrieval

To investigate set size effects in memory retrieval, we developed a model incorporating three assumptions that we consider essential for capturing retrieval in cued recall. Rather than advocating for a specific theoretical framework (e.g., an evidence accumulation process or the temporal context model), our goal was to provide mechanistic insights into memory search by adopting a minimalist approach. The three assumptions we incorporate are as follows: (1) retrieval is guided by similarity, (2) memory search is a sequential process, and (3) activation or sampling probability scales with set size. Assumption (2) (the sequential process) is particularly important, as it allows us to explicitly model why RTs increase with set size. In the following section, we introduce the model and its predictions for the three experiments we present in this paper.

To implement these assumptions, we created a sequential sampling model inspired by the assessment of retrieval completion ((ARC)-REM) model for cued-recall proposed by Diller et al. (2001); how this model differ from ours will be discussed in more detail in the alternative models section). Since our model builds on the notion of a high-dimensional, semantically meaningful memory space, we termed our model the similarity-based sequential sampling model of memory retrieval or short the SimSS model.

**Fig. 1:**
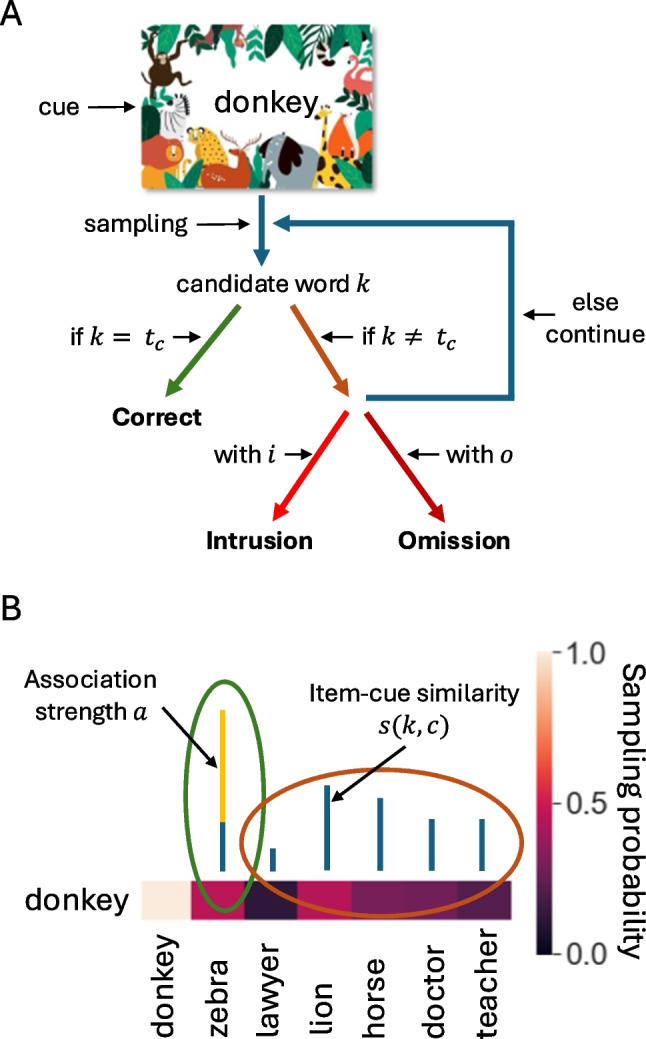
Depicted is a schema of the model. **A** The sampling process. The bolded words represent end states of the search. **B** A schematic of the sampling probabilities given a cue (donkey). The green circled word bar is the probability of sampling the correct word and all brown circled bars combined are the probability of sampling an incorrect word. The words are examples from experiment 3

The SimSS model is a model of cued recall. In cued recall, a cue is associated with a target and participants have to retrieve the correct target when later presented with a given cue. Because the sampling is based on semantic proximity, in our model, the cue can be seen as the start of the search process and memory items are inspected sequentially one by one. Which memory item is inspected is probabilistically determined by the similarity between the item and the cue. This means that more similar items are more likely to be retrieved. In the case examined here, all stimuli are words. The retrieval process proceeds as follows: When a cue is presented, the model computes the pair-wise similarity between the cue and all other items in memory (see Fig. 1B). Based on these similarities, it then samples a potential target word as shown in Fig. 1A.

If the sampled item is the target, the model accepts the target and ends the search, resulting in a correct response (green arrow in Fig. 1A). We therefore make the explicit assumption that the accuracy of recognizing the sampled item as the target is perfect. Although being an approximation, recognition from LTM has been shown to be indeed excellent in many cases (e.g., Brady et al., 2008).

If the sampled item is not the target, the model does one of three things:

1. With intrusion probability $i$, the model accepts the sampled item and ends its search, resulting in the retrieval of an intrusion (light red arrow in Fig. 1A).
2. With omission probability $o$, the model rejects the item and ends the search, resulting in an omission (dark red arrow in Fig. 1A).
3. If none of the above actions occurred, the model continues its search by taking another sample (blue arrow in Fig. 1A that loops back to sampling).

This implementation means that both error responses (intrusions and omissions) are independent of the actually sampled item and its similarity to the cue, as long as this item is not the target. Regardless of the resulting response, the model response time is defined by the number of sample steps the model takes until the search has ended (meaning a correct response, an intrusion, or/and omission is made). This time can later be transformed into RTs (e.g., by a linear transformation). For the predictions, we forewent this step.

To obtain semantically meaningful word representations, we used pretrained Word2Vec embeddings (Mikolov et al., 2013), specifically those provided by the en_core_web_md model in the spaCy Python package. The pair-wise semantic similarity between an item $k$ and a cue $c$, denoted $\text {sem}(k, c)$, is computed as the cosine similarity between their respective embedding vectors. We validated this similarity measure with human pair-wise similarity ratings in a separate study (*r* = 0.81, see Appendix A). To account for the learned association between the cue $c$ and the target $t_c$, we introduce an association strength parameter $a$, which is added to the semantic similarity (yellow line in Fig. 1B). This parameter has the same value for all learned associations. The resulting *total* similarity (accounting for both the semantic similarity and if applicable the cue-target association) between item $k$ and cue $c$, denoted ($s(k, c)$), is given by the following:

1 $$\begin{aligned} s(k, c) = {\left\{ \begin{array}{ll} \text {sem}(k, c)^\beta + a & \text {if } k = t_c, \\ \text {sem}(k, c)^\beta & \text {otherwise,} \end{array}\right. } \end{aligned}$$

where $\beta$ modulates the effect of semantic similarity on the total similarity. When $\beta =0$, the total similarity is fully defined by the association strength between cue and target. As $\beta > 0$ increases, however, the effect of semantic similarity on *total* similarity becomes relatively stronger.

The probability of each item $p(k)$ being sampled is then determined by the following:

2 $$\begin{aligned} p(k) = \frac{s(k, c)}{\sum _{j=1}^{N} s(j,c)} \end{aligned}$$

where $N$ is the total number of items in memory (including the target, but excluding the cue).

In the task we use here, participants learn several lists. However, for the sake of simplicity, the model’s memory only includes the current list. This implementation implicitly assumes that list context can constrain memory search to items from the same list. To test this assumption, we also fit a version of the model that includes words from previously learned lists (see Model results section after the experiments).

**Fig. 2:**
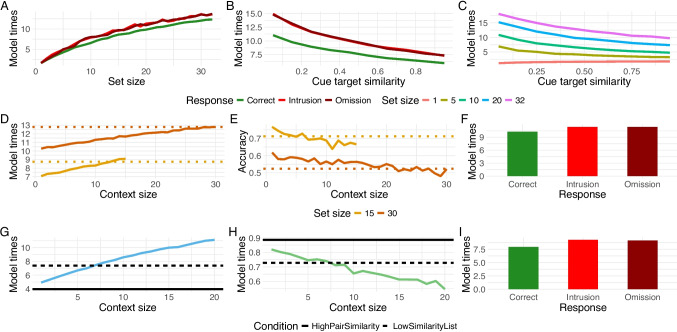
Depicted are average predictions across all simulation runs of the SimSS Model. The first, second, and third rows show the predictions for the first, second, and third experiments, respectively. **A** The locally weighted regression for the set size effect on RTs and **B** the mean values for the similarity between the cue and the target word pair. The similarities where binned into 10 bins for the averaging. Both plots also show the prediction that wrong responses will produce longer RTs. **C** The predicted interaction between similarity and set size, namely that retrieval from longer lists will be affected more by a larger cue target similarity. **D** and **E** The mean values for the context size effect on RTs and accuracy. The dotted lines in **D** and **E** represent the average performance for lists of the same size without an experimentally manipulated context. **G** and **H** The same for Experiment 3. Here, the black dotted and solid lines represent simulations of the two control conditions. **F** and **I** The faster RT prediction for correct responses in Experiments 2 and 3, respectively

We used our model to generate qualitative predictions for all three experiments. The values of the independent variables we used in the simulations were informed by experimental considerations; while these will be discussed in more detail in the respective experimental sections, we provide a brief overview here.

Experiment 1 examined how RTs and accuracy are affected by increasing set size and whether higher semantic similarity facilitates retrieval. Accordingly, we simulated set sizes ranging from 1 to 32, with each set size being repeated 1000 times. Since we did not use any actual words for the simulation, the similarity between each word and the cue was drawn from a uniform distribution between 0 and 1. The results are shown in Fig. 2 A–C.

Experiment 2 explored the effect of visual context and additionally attempted to replicate the set size effect from Experiment 1. We simulated two set sizes (15 and 30) and varied the context size within each set as in the actual Experiment (from 0 to 15 or 30, respectively). Again, each combination was simulated 1000 times. Results are shown in Fig. 2D–F.

Experiment 3 investigated the impact of semantic context, especially in comparison to control conditions that had the same set size but no coherent semantic context. This allowed us to judge when the semantic context is helpful or harmful. For this simulation, we used a fixed set size and varied the context size from 0 to 20. We also included two control conditions: one in which cue-target similarity was high ($sem(t_c,c)>0.7$), but all word pairs where dissimilar to each other ($sem(k,c)<0.3$ if $k \ne t_c$) and one where all words (including the words within a pair) were dissimilar to each other ($sem(k,c)<0.3$). Each condition was simulated 1000 times. The results are shown in Fig. 2G–I.

Across all simulations, we used the same model parameters: ($a$ = 0.2, $o$ = 0.02, $i$ = 0.02, $\beta$ = 1). In Appendix D, we show that the effects we predict with these parameter values are robust within a reasonable range of parameter values. To represent an increased similarity between cue and target for simulations of the context (simulations for Experiments 2 and 3), we added a constant value $d$ to the pair-wise similarity of all items belonging to the same context. This means the following update:

3 $$\begin{aligned} s(k, c) = {\left\{ \begin{array}{ll} s(k, c) + d & \text {if } k \text { shares context}, \\ s(k, c) & \text {otherwise,} \end{array}\right. } \end{aligned}$$

*d* was set to 0.3 for these simulations, and *s*(*k*, *c*) is calculated as shown in Eq. (1).

We specify our experimental hypotheses in the respective sections, but here is a brief summary of the model’s general predictions: Larger set sizes lead to higher RTs, particularly for incorrect responses (see Fig. 2A). An effect of cue-target similarity, leading to faster and more accurate responses for more similar cue-target similarities, interacts with set size (see Fig. 2B and C). Incorrect responses tend to be slower than correct ones (see Fig. 2A, B, F, and I). Introducing a context that is associated with a subset of items affects both RTs and accuracy, with the effect depending on context size.

While we view the sampling part of the model as representing a cognitive process that people engage in, i.e., a cognitive process in the sense of a process model (Borsboom et al., 2003), it is important to note that because of the simplified nature of how the model accepts and rejects samples, the model can also be viewed as a measurement model.

### Alternative Models

While our model emphasizes key components such as similarity, sequential search, and the effect of other items on the probability of retrieving the target with a strong focus on the modeling of RT, other models offer different perspectives on these processes. To contextualize our approach within the broader landscape of memory models, we compare our model with three alternative approaches, the ARC-REM model for cued recall (Diller et al., 2001), the Source of Activation Confusion (SAC) model (Popov & Reder, 2020), and the Temporal Context Model (TCM) introduced by Howard and Kahana (2002a). This comparison helps to clarify how our model aligns with and deviates from other theoretical perspectives in the study of memory retrieval.

As mentioned, our model builds on the (ARC)-REM model for cued-recall proposed by Diller et al. (2001), which is an extension of the search of associative memory (SAM) model (Raaijmakers & Shiffrin, 1981, 1980) and the retrieving effectively from memory (REM) model (Shiffrin & Steyvers, 1997). In fact, it can be viewed as one implementation of these models. For the sake of this comparison, we will focus on the model proposed by Diller et al. (2001), because of its focus on RT and its use of vector-based representations. The ARC-REM model proposes that cued recall can be viewed as a sequential search: a memory image is sampled based on likelihood ratios and is then accepted or rejected. If accepted, recovery (meaning the retrieval of the image into conscious memory) is attempted. If recovery fails or the image is rejected, the model decides whether to abort the search. A response is made if the image is recovered or the process is aborted; otherwise, sampling continues. Our implementation modifies and simplifies this model in a few key ways. (1) In the ARC-REM model, the likelihood ratios are calculated using incomplete and error prone vectors of 20 values, which represent learned words. In our model, we ignore that encoding can be error prone (in part, because we motivate good learning by letting participants recall a learned pair twice in the learning phase and in part as a simplification, because we want to focus on modeling recall). Furthermore, to emphasize the assumption that memory retrieval operates on a semantic network, we represent words not through arbitrary values, but through pretrained Word2Vec embeddings that encode semantic information about a given word. Consequently, where ARC-REM computes likelihood ratios from cue-image matches, we derive sampling probabilities from the semantic similarity between cue and target (via Word2Vec) and an association strength reflecting the learned cue-target pairing. (2) In ARC-REM, image acceptance and sampling abortion decisions depend on likelihood ratios and a familiarity signal. In contrast, the SimSS model uses fixed parameters, $i$ (intrusion probability) and $o$ (omission probability), that are independent of the sampled image, except when the correct word is sampled, which is always accepted. We justify this simplification by assuming recognition is more reliable than recall. Additionally, while ARC-REM increases acceptance probability as a response deadline approaches (Nobel & Shiffrin, 2001), we omit this adjustment due to the unbounded recall window in our design. (3) Since we do not use an error prone encoding process, there is no way in which the recovery of an image/memory can fail. (4) ARC-REM assigns different durations to each stage of a sampling step, with the first step taking extra time to account for full feature activation, primarily to model RTs in recognition tasks. In SimSS, we simplify this by using a constant time increment per sample. In fact, for our predictions, we do not convert sample steps into RTs at all. In summary, these differences highlight that our model is a streamlined version of ARC-REM with a key addition: semantic similarity-based sampling, which is absent in the original. This allows us to reduce the number of parameters from 20 to just 4 and simplify the model fitting, while preserving the core idea of sequential sampling in cued recall (Shiffrin, 1970; Raaijmakers & Shiffrin, 1980, 1981; Diller et al., 2001). We take this minimalist approach, to test if the qualitative hypotheses we generate with just a few assumptions match the results of our experiment. We later investigate if a quantitative fit of the model can be improved by further assumptions.

A second model, which serves as a relevant comparison, is the SAC model. Here, we will focus on the extension introduced by Popov and Reder (2020). In general, the SAC model builds on the spreading activation theory (Collins & Loftus, 1975), in which memories are represented in local nodes and the activation spreads through connections (edges), creating a network. When we first built our model, we considered this framework as a potential alternative to the vector-based representations we ended up using for our sequential search process. In SAC, recall depends on the base-level strength of a node, which in turn depends on its activation history. In cued recall, the cue node (and the context node) gets activated and this activation spreads to all connected nodes, depending on three factors: (1) the base-level strength of the cue node (which determines the nodes own activation), (2) the strength of the connection between the cue and target node, and (3) the total strength of all connections the cue node has. Specifically, a node can only activate other nodes based on its own activation. The degree to which a node activates connecting nodes depends on the strength of the connection divided by the strength of all connections the node has. That principle is very similar to how we calculate $p(k)$. The outcome of the memory retrieval in the SAC model depends on whether the activation of a node passes a retrieval threshold. Another helpful comparison here is that if you have learned, e.g., the word pair donkey-zebra, and you get the cue donkey, what you retrieve/activate is not the semantic node for zebra, but instead the episode of the word pair. In a way, SimSS represents this episode with the association strength, but each word is still represented separately. As such, episodes do not exist in our model, beyond the features of an episode, which increase the similarity between items. As we mentioned, we built our model with three key assumptions about memory in mind: (1) similarity matters, (2) memory retrieval in cued recall is a sequential search process, and (3) there needs to be a mechanism that scales activation or the probability of sampling potential target items given the number of learned items. The first assumption is not part of the SAC model presented by Popov and Reder (2020), but could be easily implemented in the connections between nodes (De Deyne et al., 2019). The third assumption is indeed present in the calculation of the activation, which is spread from one node to the next. However, while this would result in similar accuracy predictions, the SAC model lacks an explicit process, by which RTs are generated and does not propose a sequential search, but instead an activation process which could predict constant RTs without further clarifications or assumptions. This was also the reason why we did not use the spreading activation theory for our modeling approach.

While the TCM model is a computational model of episodic memory retrieval, it has primarily been used to model free recall. The model was first introduced by Howard and Kahana (2002a) and proposes that memory functions around a continuously drifting internal context representation, which supports recall through associations with studied items. As such, the model describes a set of rules by which the context gets updated. Items are recalled by using the current context as a cue. Recalled items then update the context, promoting recall of temporally or semantically related items. One striking similarity between the TCM and the SimSS model lies in how items are sampled. The main difference here is that we use the similarity between cue and an item $s(c,k)$, and the TCM uses the activation of each item (which is driven by the similarity to the current context) to calculate the sampling probability (meaning the probability of recalling an item). In some ways (at least when it comes to accuracy), the SimSS model can be viewed as a special case of the TCM, where the context does not drift, but has strict boundaries (i.e., a word is associated with a cue or it is not, or a word is associated with a visual context or it is not), and the currently activated context is mainly driven by the cue word. With these simplifications, the SimSS model (in its current form) cannot predict contiguity effects or other more nuanced temporal dynamics, but these are also less likely to play a role in cued-recall than free-recall. However, there are also clear differences. The main difference is that the TCM does not assume that a memory, once sampled, would be rejected; a word either is recalled or not. Consequently, the reaction times are either not modeled or need an additional process to be estimated. Furthermore, similarity is not part of the base TCM, but adaptations like the one by Morton and Polyn (2016) show that similarity can be included in the model. Interestingly, in this study, they found that while the retrieved temporal context cues the next retrieval, when it comes to semantic cues, not the context, the semantic associations of the item itself serve as a cue for the next retrieval. This result might support the idea of the item-based semantic search we used here (meaning all similarities we use for the search are based on pair-wise similarities to the cue).

In summary, other modeling approaches, whether similar or different in their implementation, can produce comparable predictions, particularly in terms of accuracy. In addition, many other models could be considered candidates for modeling retrieval. Our decision to use the SimSS model is motivated by its focus on reaction times and its use of a minimal set of theoretically relevant assumptions. This allows us to generate testable hypotheses without committing to a specific underlying cognitive process.

## Experiment 1: Set Size Effects on Memory Retrieval

In this experiment, we studied how retrieval times and accuracy of cued-recall from LTM are affected by the number of learned word pairs. We also investigated whether participants use the semantic similarity of a word pair to improve retrieval speed and accuracy.

### Methods Experiment 1

#### Design

In this experiment, participants learned lists of word pairs, which they later had to recall in a cued recall task, in which we used one word of a word pair as the cue. The two phases were separated by a letter span task, which ensured that working memory was not used for the recall. For the cued-recall task, we manipulated two factors: set size and similarity of the words in a word pair. To ensure that we could capture the functional form of the set size effect, we included 16 set size levels (2–32 word pairs in steps of two). Because this resulted in too many word pairs for a single participant to learn, but we also wanted to look at the set size effects across blocks, we created four combinations of four set size each, which were varied between subjects. As a result, each participant was exposed to four different set sizes (A, [2, 12, 22, 32]; B, [4, 14, 24, 26]; C, [6, 16, 18, 28]; D, [8, 10, 20, 30]). We predefined these set sizes to control for the total number of word pairs each participant learned (68 word pairs). This set up ensured that all set sizes were presented an equal number of times and that each participant had seen the same number of word pairs. Similarity between word pairs was manipulated within-subject. We measured both reaction times and recall accuracy as outcome variables. In the letter span task, we manipulated the number of letters (3–7) within-subject to have a letter span measure for potential follow up questions. Accuracy was measured as an outcome variable.

**Fig. 3:**
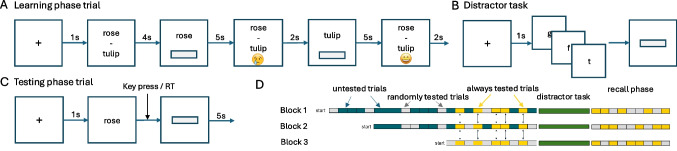
Illustration of the trial structure in Experiment 1. **A** One trial from the learning phase. The numbers above the arrow indicate how long the image left of the arrow was presented. **B** One trial from the letter span task, which we used as a distractor task. The number of sequentially presented letters varied. **C** One trial from the testing phase. **D** A schematic of the proactive trial structure we used in Experiment 1 to control for lag effects

#### Participants

Based on a pilot study with 14 participants, we estimated that 120 participants (after exclusion) would be needed to reliably detect a set size effect if the effect observed in the pilot was true. Accordingly, we collected data from 174 adults (81 female, 4 non-binary or unknown, age range 18 to 45, mean = 32.77; SD = 6.95) via Prolific (www.prolific.com). We excluded participants who did not provide at least two valid responses (valid responses for this analysis are described in the analysis section) for each list (58 participants) and participants who reported that they wrote down the word pairs as a memory aid during the task (1 participant). Both of these exclusion criteria were preregistered and led to the inclusion of 115 participants. Participants were paid up to $12.00 ($6.00 base payment plus a maximum bonus of $6.00; Mean = 10.25; the bonus was calculated by multiplying the maximum bonus with the average accuracy in the cued-recall and the letter sequence tasks, i.e., if a participant got 90% of the letter sequence task trials and 70% of the cued-recall trials correct they received a bonus of (0.9 + 0.7)*0.5*$6 = $4.8). To ensure that the task was understood correctly, all participants had to answer three comprehension questions before starting the experiment. Most participants finished the experiment in under 50 min (including breaks, which could be taken after each block). Informed consent was obtained from all participants before the experiment started. This and all following studies were approved by the ethics committee of the medical faculty of the University of Tübingen (number 701/2020BO).

#### Materials

To control for variability in RTs or accuracy due to word length (Katkov et al., 2014), we used words with a length of four to six characters in the cued-recall task. All words were taken from the labels of the THINGS dataset (Hebart et al., 2019) and were proper nouns. To manipulate the similarity between the words within one pair, we created word pairs that fell into five bins of similarities (0.0–0.2, 0.2–0.4, 0.4–0.6, 0.6–0.8, and 0.8–1.0). We used the cosine similarity between the pre-learned Word2Vec embeddings (Mikolov et al., 2013) we introduced in the modeling section. Each list with a set size larger than 5 contained an equal number of word pairs from each of the bins, to ensure that all lists had a similar average similarity and that all participants saw the same number of pairs from each similarity bin. If the list length was not divisible by 5, the remaining word pairs were randomly chosen. The same was done for lists of length below 5.

#### Procedure

Participants were instructed to recall the word pairs (for the cued-recall task) and the letters in order of presentation (for the letter span task) as fast and accurately as possible. We emphasized accuracy by telling participants that their bonus depended on the percentage of correct responses but not on the speed at which they responded. In total, each participant completed four blocks with different set sizes (see design section), which were presented in random order. Each block consisted of three phases. In all phases, each trial started with a fixation cross which was presented for 1 s. In the first phase, the learning phase (see Fig. 3A), participants were presented with a sequence of word pairs in the center of the screen. Because we wanted participants to achieve a high recall accuracy (Ratcliff & Murdock, 1976; Shiffrin, 1970) for our RT analysis each pair was presented for 4 s, followed by a short learning phase. Here, one of the words was replaced with an empty response field and participants had 5 s to type the omitted word. The word pair was then presented again for 2 s together with a happy or sad smiley indicating whether the response was correct or not. We repeated the same procedure for the other word pairs. This extended study procedure was also meant to minimize reduced attention effects and displaced rehearsal as a confounder in longer lists (Kinnell & Dennis, 2011) through increased engagement with the current word pair.

To ensure that participants recalled the words from LTM, we implemented a letter span task, which requires working memory, as a distractor task (Daneman & Carpenter, 1980) in the second phase of each block (see Fig. 3B). This also served to prevent chances for rehearsal, which would favor shorter lists (Kinnell & Dennis, 2011). Here, participants were sequentially presented with 3–7 letters, which they then had to recall by typing in the letters in the presented order into a response field. Each letter was presented for 1 s and participants had to provide their response within 10 s. If the response was correct a happy smiley and if not a sad smiley was presented for 1 s. Each letter span task consisted of 10 trials with each of the five lengths presented twice in random order. To avoid the accidental formation of words, all letters were consonants (f, h, j, k, l, n, p, q, r, s, t, y). No letter appeared twice within the same sequence.

In the last phase, the cued-recall phase (see Fig. 3C), participants were presented with some of the words from the first phase. Their task was to recall the not-presented word of a word pair (the target) given the presented word (the cue). As soon as they knew the word to be recalled, they were instructed to press the space bar. The elapsed time since the presentation of the cue was our measure of the retrieval time. To prevent premature key-presses, participants only had 5 s to type their response after they had pressed the space bar. To ensure that slow typing was not an issue and to minimize the effect of typos on the feedback, participants only had to type the first three letters of the target word correctly. If participants could not recall the target, they were asked to respond by typing “0.” Each response was followed by feedback. We included the feedback to keep up the motivation throughout the study (Reinecke & Gajos, 2015). Which word of a word pair was the cue was always determined randomly. To control for lag (retention interval) as a confounder, we always cued words from the same positions (2, 4, 5, 7 and 9) counting from the back of the list (i.e., a proactive design Kinnell and Dennis, 2011). This design in combination with the distractor task also helps control for contextual reinstatement as a confounder for set size effects, because the list context should be similarly strong for all conditions at the time of recall. Each of the 5 tested words came from a different similarity bin. To prevent the participants from learning which positions got cued, 5 other words were randomly cued, resulting in 10 cued words in total, which were presented in random order (see Fig. 3D) for a schema of this). For sets smaller than ten word pairs, participants had to recall all the word pairs they learned in phase one in a random order.

#### Hypotheses

We based our hypotheses on the predictions of the similarity-based sequential sampling (SimSS) model. The SimSS model is a model of the recall process and only simulates the test phase. Based on this model, we hypothesized the following: (1) RTs in the cued-recall task increase with the length of the list (i.e., the set size) for correct responses (see Fig. 2A). (2.A) Targets that are similar to the cue can be retrieved faster (see Fig. 2B), (2.B) especially for longer lists (i.e., we expect a negative interaction between set size and semantic similarity between cue and target, meaning that similarity decreases RTs more for longer lists (see Fig. 2C). (3) RTs are longer for incorrect responses (see Fig. 2A and B).

All hypotheses were preregistered (https://osf.io/uqjn3).

#### Analysis

We statistically tested the three hypotheses with these three models (also preregistered):

1. RT $\sim$ set size + (1 + set size | participant)
2. RT $\sim$ set size * similarity + (1 + set size + similarity | participant)
3. RT $\sim$ accuracy + (1 + accuracy | participant)

Similarity refers to the similarity between cue and target. For the first and second RT analysis, we only included correct responses. Furthermore, we excluded all RTs that exceed 20 s (e.g. to exclude trials during which participants left the screen). We also only used RTs from the 5 positions that were cued in all lists that had a minimum of 10 word pairs. Participants who did not provide us with at least 2 valid RTs that met these inclusion criteria for each of the set sizes were excluded from this analysis. For the accuracy analysis, we included all responses with RTs below 20 s. For the sake of completeness, we also analyzed set sizes smaller than 10, which we could not control for lag. In this analysis, we included all RTs for correct responses below 20 s from all set sizes (i.e., not controlled for lag).

**Fig. 4:**
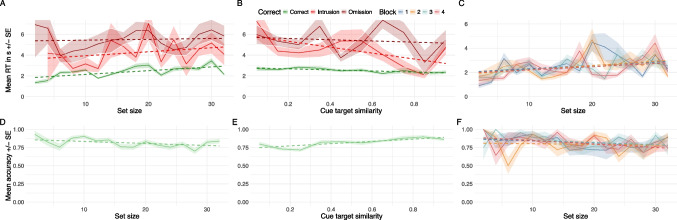
Results Experiment 1. **A**, **B**, and **C** The mean RTs (solid lines) and regressions (dotted lines) of the RTs. The shaded are represents the standard error. **A** and **C** The RTs across set sizes. In **A**, the colors represent the correctness of the response. In **C**, the colors represent the different blocks. Here, only correct responses are plotted. **D**, **E**, and **F** The same, but for accuracy. All shaded areas around the mean represent the standard error (SE) of the mean

We implemented the models as Bayesian hierarchical linear regressions using the *brms* package (Bürkner, 2017, 2018) in the R statistics environment (R Core Team, 2024). We used a lognormal Likelihood function in these models, because the models did not converge when using an exGaussian (as preregistered). For the models that had accuracy as the outcome, we used a Bernoulli likelihood function. All models used default priors. To estimate whether the predictors affected the RTs as hypothesized, we used Bayes factors (BFs) (Kass & Raftery, 1995). Specifically, we performed model comparisons using maximally structured mixed effects models (Barr et al., 2013). This means that we always compared the full models specified above against models that did not contain the target variable as a fixed effect, but did still have it as a random effect. If the full model does not explain the data better than the model excluding the target variable, then the target variable is unlikely to have a strong and systematic contribution to the change in RTs. We used *bridge sampling* (Gronau et al., 2017) as included in the *brms* package to approximate BFs for these comparisons. The Bayes factor (BF) is defined as the ratio of the marginal likelihoods of the two competing models: $BF_{10}$=$\frac{p(data|H_1)}{p(data|H_0)}$. It quantifies the relative evidence provided by the data for one hypothesis over another, given the hypotheses are equally likely a priori. A BF larger than 1 provides evidence for the alternative hypothesis ($H_1$), while a BF below 1 provides evidence for the null hypothesis ($H_0$). For example, a BF of 2 indicates that the data are twice as likely under the alternative hypothesis. Generally, BFs larger than 3 are interpreted as providing substantial evidence for one hypothesis over the other (Jeffreys, 1961). All BFs are reported in favor of the alternative hypothesis ($BF_{10}$). All models we used to estimate effects are linear regression models. We sampled from the posterior distributions of the model parameters using 4 independent Markov chains (each with 5000 warm-up samples and 5000 stored samples). We tested the convergence of the chains by means of the $\hat{R}$ values and by visual inspection. All $\hat{R}$ values were equal to 1, and chains mixed well covering the target distributions. In all analyses, set size and similarity were mean centered and *z* standardized.

### Results Experiment 1

As hypothesized, RTs increased with set size ($\hat{\beta }=0.09$, 95% HDI = [0.05, 0.13], $BF_{10} > 100$, see Fig. 4A). On average, increasing set size by one pair (e.g., from 2 to 4 or 8 to 10), slowed down the response by 0.06 s. Increased similarity between the cue and the target resulted in faster RTs ($\hat{\beta }=-0.06$, 95% HDI = [$-0.08$, $-0.04$], $BF_{10} > 100$). On average, the responses were 0.68 s faster if the similarity was above 0.8 when compared to similarities below 0.2. However, there was no evidence for the similarity *x* set size interaction ($\hat{\beta }=-0.01$, 95% HDI = [$-0.03$, 0.01], $BF_{10} < 0.01$), supporting hypothesis 2.A, but not 2.B (see Fig. 4B). We observed evidence for Hypothesis 3, with RTs for wrong responses being on average 2.8 s slower than RTs for correct responses ($\hat{\beta }=-0.76$, 95% HDI = [$-0.85$, $-0.68$], $BF_{10} > 100$, see Fig. 4A and B). We also post hoc tested if the RTs differed for the different responses by splitting the wrong responses into omissions and intrusion. We found that both omissions ($\hat{\beta }=0.91$, 95% HDI = [0.81, 1.01]) and intrusions ($\hat{\beta }=0.56$, 95% HDI = [0.43, 0.67]) have longer RTs than correct responses ($BF_{10} > 100$) and that omission are on average 0.87s slower than intrusions. In addition to RTs, Hypotheses 1 and 2 were also supported when analyzing accuracies (set size: $\hat{\beta }=-0.26$, 95% HDI = [$-0.42$, $-0.04$], $BF_{10}$ = 4.67, see Fig. 4D and similarity: $\hat{\beta }=0.36$, 95% HDI = [0.18, 0.55], $BF_{10} > 100$, see Fig. 4E). The results remained the same in the analysis including responses from smaller set sizes and not controlling for lag.

To investigate whether the list context can be used to constrain the search set, we also tested the effect of block. The idea being that after presentation of the pairs in the 4th block, all participants had observed the same number of pairs. Accordingly, if participants could not use the list/block context to constrain their memory search, the set size effect should increase over blocks, or in other words, there should not be a set size effect in the last block any more. To test this, we first looked at whether there was an interaction between block and set size or a main effect of block in the lag controlled RTs.^1^ However, there was no interaction ($\hat{\beta }=-0.02$, 95% HDI = [$-0.07$, 0.03], $BF_{10} < 0.1$) or main effect of block ($\hat{\beta }=-0.01$, 95% HDI = [$-0.05$, 0.03], $BF_{10} < 0.01$). To test whether there was a difference in mean reaction times (RTs) between Blocks 1 and 4, a Bayesian paired-sample *t*-test using the BayesFactor package (Morey et al., 2015) was conducted. We observed evidence in favor of the null, i.e., that there is no difference between the RTs in Block 1 and 4 ($BF_{10}$ = 0.31). The same was true for accuracy ($BF_{10}$ = 0.21). Both these exploratory analyses support the conclusion that the set size effect is constrained to a specific list and that it does not accumulate over several lists presented in the course of the experiment (see Fig. 4C).

Furthermore, to make sure that the effects we found were not affected by word frequency, which has been shown to strongly effect encoding and retrieval in LTM (e.g. Popov & Reder, 2020), we added the SUBTLEX word frequency count of the target word as a main effect both to the full RT model and the full accuracy model. Word frequency of the target did neither affect the recall speed ($\hat{\beta }=-0.02$, 95% HDI = [$-0.04$, 0.01], $BF_{10} < 0.1$) nor the accuracy ($\hat{\beta }=0.24$, 95% HDI = [$-0.01$, 0.72], $BF_{10}$ = 1.3). Including the frequency did not change the effects of similarity (RT, $\hat{\beta }=-0.06$; accuracy, $\hat{\beta }=0.38$) and set size (RT, $\hat{\beta }=0.12$; accuracy, $\hat{\beta }=-0.23$) strongly.

### Discussion Experiment 1

We observed that both RTs and accuracy were negatively affected by set size. Semantic similarity between cue and target made recall more accurate and faster, regardless of set size. Furthermore, RTs were slower for incorrect responses. These results support the idea that memory retrieval builds on a sequential search through a semantic network. Additionally, we found that the list context was relevant for the set size effect, with the set size effect not accumulating across lists. This strongly suggests that participants can use the list context to constrain the items they consider. In other words, temporal context is an important organizational dimension in LTM (Brown et al., 2007). In Experiment 2, we tested whether additional, task-irrelevant cues that are associated with only a temporally dispersed subset of the word pairs can be used similarly as a temporal context to aid memory retrieval. Therefore, we manipulated the number of stimuli associated with a visual context cue, which have previously been used to successfully study context effects (e.g., Liu et al., 2024; Verkoeijen et al., 2004; Racsmány et al., 2021).

## Experiment 2: Effect of Visual Context Cues on Memory Retrieval

In this experiment, we studied how the number of learned word pairs that are associated with a visual cue which was not associated with the task beyond the fact that it was present both during encoding and recall affects retrieval times and accuracy of cued-recall from LTM.

### Methods Experiment 2

#### Design

We used the same general three phase setup as in Experiment 1. For the cued-recall task, we manipulated two factors: (1) context size, i.e., the number of word pairs associated with a visual context cue, and (2) set size. Set size was either 15 or 30 word pairs and was manipulated between-subjects. To get a roughly equal number of words, the set size 15 conditions had 5 blocks and the set size 30 conditions 4 blocks. Context size was manipulated within and between subjects, with some context sizes always shown, to ensure we had enough data for smaller context sizes (3–27 and 5–25 for the 30 and 2–13, 3–12, 4–11 for the 15 set size condition). To get a baseline accuracy and RT measure for each set size, we also had one block without context cues. The last block for each condition was a random context size varied across participants. We measured both retrieval times and accuracy as outcome variables. The letter span distractor task was the same as in Experiment 1.

#### Participants

We collected data from 151 adults (74 female, 2 non-binary or unknown, age range: 18 to 45, mean = 32.88; SD = 6.83) via Prolific. To ensure that the task was understood correctly, all participants had to answer three comprehension questions before the start of the experimental trials. We excluded 27 participants who provided fewer than three correct responses (with RTs below 20 s) for the smaller context sizes (i.e., context sizes up to 5) and 5 participants who used memory aids (criteria again preregistered). This left us with 119 participants (58 female, 1 non-binary or unknown, mean age = 32.7; SD = 6.55), 59 in the set size 30 condition and 60 in the set size 15 condition. Participants were paid up to $14.50 ($7.50 base fee plus a maximum bonus of $7.00; mean = 6.64) or $18.00 ($9.00 base fee plus a maximum bonus of $9.00; mean = 7.75), depending on whether they were in the set size 15 or 30 condition. Most participants finished the experiment in under 60 min and 80 min, respectively (including breaks, which could be taken after each block). Informed consent was obtained from all participants before the experiment started. The study was approved by the ethics committee of the medical faculty of the University of Tübingen (number 701/2020BO).

#### Materials

For the cued-recall task, we used words from the same source as in Experiment 1. The 8 visual context cues that were presented with the word pairs were taken from www.freepik.com and randomly chosen for each context size. No participant saw the same context cues twice.

#### Procedure

Participants were instructed to try to recall the word pairs (for the cued-recall task) and the letters (for the letter span task) as fast and accurately as possible. Participants were told that their bonus depended on the percentage of correct trials, but not on the speed at which they responded. In total, each participant completed five (set size 15 condition) or four blocks (set size 30 condition). Each block consisted of the same three phases as in Experiment 1, with two differences. First, because the set size effect was not the main interest of this experiment, all lists a participant experienced were of the same length (15 or 30 word pairs, for the two groups, respectively). We still used two set sizes to ensure that any potential context size effect generalizes to a different set size, and to replicate the set size effect from Experiment 1. 2. In the recall phase, we queried all words in a random order, since with constant set sizes, lag should on average be the same for the different context size conditions and should therefore no longer be a confounder.

To measure the effects of context size on retrieval times and accuracy, we varied the number of word pairs associated with each visual context cue as well as the position of the word pair on the screen (each context had a fixed position associated with it). Each participant had one control block without context cues (i.e., a white background). The remaining blocks always contained two contexts, with randomly assigned visual cues for each participant. The order of word pairs was random within block and the order of blocks was random for each participant. To control for similarity of the word pairs, we only used word pairs with a similarity between 0.45 and 0.55. The rest of the procedure, i.e., the presentation times and the three phases (learning, distraction, and recall), were the same as in Experiment 1.

**Fig. 5:**
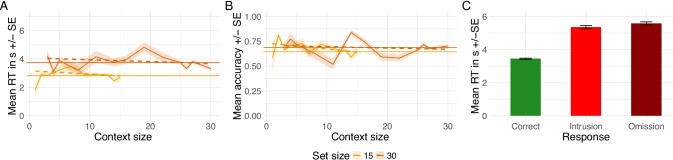
Results Experiment 2. **A** The effect of visual context on RTs. The different colors represent the two set sizes and the solid line shows the mean RT of the control condition, in which no visual context was presented. **B** The effect of visual context on accuracy. The solid lines show the mean accuracies of the control condition, in which no visual context was presented. **C** The mean RT for the different response types. All shaded areas around the mean and the error bars represent the standard error (SE) of the mean

**Table 1:** Control conditions, Experiment 3

	*List similarity*	
*Pair similarity*	High	Low
High	Large context size manipulation	High pair similarity control condition
Low	Not implemented	Low list similarity control condition

This illustrates the logic behind the control conditions in Experiment 3

#### Hypotheses

Using the SimSS model, we hypothesized the following: (1) The more word pairs associated with a context cue, the longer it takes to retrieve the target (see Fig. 2C). In comparison to a control condition with no context, the SimSS model predicted detrimental effects of larger contexts. To see if these effects exist in humans, we formulated three exploitative hypotheses: 2. Exploratory: Are the RTs for larger context sizes slower than the RTs if no context cue is present (given the same set size)? 2. a) Unspecific context cues are detrimental: RTs for larger context sizes are slower than the RTs if no context cue is present. 2. b) Context cues are always helpful: the smaller the context size, the larger the benefit for the RTs. If the context size is the same as the set size there is no benefit. 2. c) Hypothesis 2. a) is true, but if a context cue starts becoming detrimental, we are able to ignoring it, resulting in a lack of context effects for larger context sizes.

Replication from Experiment 1: 1. RTs in the cued-recall task increase with the set size for correct responses (see Fig. 2D). 2. RTs are slower for incorrect responses than for correct responses (see Fig. 2F)

All hypotheses were preregistered (https://osf.io/83khx).

#### Analysis

We analyzed these hypotheses with the following model (again preregistered):

1. RT $\sim$ context size + set size + (1 + context size | participant)

In the original preregistration, we included set size as a random effect, but since set size was only manipulated between subjects, we removed that random effect from the fitted model. We again excluded incorrect responses (for the RT analysis), responses with RTs exceeding 20 s, and additionally blocks, in which participants did not achieve an accuracy of at least 30% (all preregistered). Context size was mean centered and z standardized. Set size was treated as a factor with two levels.

### Results Experiment 2

In contrast to hypothesis 1, RTs did not change with increasing context size ($\hat{\beta }=-0.03$, 95% HDI = [$-0.05$, $-0.01$], $BF_{10}$ = 0.69, Fig. 5A), and neither did accuracy ($\hat{\beta }=-0.08$, 95% HDI = [$-0.17$, 0.01],$BF_{10}$ = 0.47 Fig. 5B)). Accordingly, we could also not investigate whether the effect of context size becomes detrimental when context size grows (hypothesis 2). Accuracy, however, did not differ between set sizes ($BF_{10}$ = 0.22). Replicating the results from Experiment 1 with Bayesian independent *t*-tests, we found that correct RTs were on average 0.88 s faster for a set size of 15 when compared to correct RTs for responses with a set size of 30 ($BF_{10}$ = 4.57) and RTs were on average 2.51 s faster for correct responses than for incorrect responses ($BF_{10} > 100$). Post hoc tests showed that this is true for both intrusions ($BF_{10} > 100$) and omissions ($BF_{10} > 100$) with correct responses being 2.08 s faster than intrusions and intrusions being 0.76 s faster than omissions ($BF_{10}$ = 3.36, see Fig. 5C).

### Discussion Experiment 2

Additional task unrelated visual context cues did not have an effect on memory retrieval. This contrasts strongly with Experiment 1, in which the effect of the list context was strong. There are several potential explanations for this difference. First, it is possible that temporal separation plays a crucial role in context effects. As such, context features that are associated with a subset of items that were all presented in a mixed fashion might not have any noticeable effect on memory retrieval. Second, it is possible that the visual, non-task related cues were not an important or attended to context feature and were not used to facilitate memory retrieval. To test these two hypotheses, we designed a third experiment, where instead of visual context cues, we used semantic categories in addition to visual category cues to investigate if the additional (semantic) cues facilitate memory retrieval. By using this stronger context manipulation, which has led to context effects more reliably (Wickens, 1970; Light & Carter-Sobell, 1970), we can test whether context features can influence memory retrieval, even when they are not temporally separated and we can also compare the effect of different context manipulations (i.e., visual vs. categorical). Furthermore, we are interested in whether the specificity of these cues (i.e., the number of items that are associated with each of these categories) matters for the helpfulness of these cues.

We were, however, able to replicate the set size effect from Experiment 1 and could also show again that retrieval of incorrect responses takes longer. Taken together, these results support the idea of a sequential search process, which we will discuss further in the modeling section.

## Experiment 3: Effect of Semantic Categories on Memory Retrieval

In Experiment 2, we manipulated the visual context and did not find that smaller contexts are helpful. Here, we used semantic properties of the cue to induce a stronger context manipulation (Wickens, 1970) to study how retrieval times and accuracy of cued-recall from LTM are affected by the number of learned word pairs that are associated with a semantic category.

We also included two control conditions without semantic category cues that allowed us to meaningfully compare the performance for the different context sizes with a baseline without context cues. To make meaningful comparisons, we manipulated the similarity of the words in these conditions. Specifically, we considered the four scenarios that occur if you fully cross the similarity between cue and target with the similarity of the cue and target to the rest of the list. This results in 4 distinct scenarios (see Table 1): First, a high similarity between all (or most) words in the list, which is the scenario we get when we have large context sizes. This results in most word pairs being from the same category (e.g., they are all animals). If the specificity of a cue matters, this should result in worse recall. The second is a scenario with a high similarity between cue and target and a low similarity between the cue and the rest of the list. If the similarity between cue and target and the specificity of the cue matter, this should be the scenario, in which it is easiest to retrieve the target given the cue, and should give an upper baseline. It also allows us to test whether these two factors matter by testing whether performance is indeed best in this condition. This scenario is similar to conditions with context sizes of one, but the separate control condition allowed us to collect a larger number of data points helpful for statistical comparisons. The third is a no semantic context scenario with a low similarity between all words in the list, which is our second control condition, and the baseline we use to test whether large contexts are worse for performance than the presence of no context. The fourth scenario would have a low similarity between cue and target and a high similarity between the cue and the rest of the list. We did not use this scenario, as it is hard to implement and does not additionally help us test our hypotheses, since the role of specificity is already being tested with the context size manipulation.

In this study, we focus both on RTs and accuracy. As sample size was limited in Experiment 3 due to budget constraints and RTs in the cued-recall task generally tended to be noisier than accuracy, we mainly focused on accuracy here.

### Methods Experiment 3

#### Design

We used the same general three phase setup as in Experiments 1 and 2. For the cued-recall task, we manipulated two factors: First, the number of word pairs associated with a semantic category (context size). The category size had 6 levels (0–20 word pairs belonging to a category), which we manipulated both within and between subjects (see procedure below for more details on this). The second factor we manipulated was the similarity of the words in the control condition, with one high pair similarity conditions, in which the words in a pair had a high similarity and each pair had a low similarity to all other pairs, and a low similarity condition, where all words were dissimilar and randomly paired together. The similarity between word pairs in the control condition was manipulated between-subjects. We measured both RT and accuracy as outcome variables. The letter span task was the same as in Experiments 1 and 2.

#### Participants

We collected data from 118 adults (59 female, 3 non-binary or unknown, age range: 18 to 45, mean = 31.42; SD = 6.4) via Prolific. To ensure that the task was understood correctly, all participants had to answer three comprehension questions before the start of the experimental trials. We excluded participants who provided no response to more than 20% of the retrieval trials (1 participant, see details below) and who reported that they wrote down the word pairs as a memory aid during the task (3 participants). Both exclusion criteria were preregistered. Furthermore, the data of 2 participants was not transmitted. This left us with 112 participants. Participants were paid up to $16.00 ($9.00 base fee plus a maximum bonus of $7.00; mean = 13.77); the bonus linearly depended on the accuracy in both the cued-recall and the letter span task, with the cued-recall task having twice as much weight as the letter span task. That is, if a participant had a recall accuracy of 90% in the letter span task and of 60% in the cued-recall task, they received a bonus of (0.6*2/3 + 0.9*1/3)*$7 = $4.9). Most participants finished the experiment in under 65 min (including breaks, which could be taken after each block). Informed consent was obtained from all participants before the experiment started. The study was approved by the ethics committee of the medical faculty of the University of Tübingen (number 701/2020BO).

#### Materials

To control for an effect of word length on RTs, we only used words consisting of 4–7 letters in the cued-recall task. We included slightly longer words than in Experiment 1 to be able to create the high similarity pairs (see Appendix C). For the same reason, we also included nouns that we gathered from other sources (e.g., our minds, ChatGPT) besides the THINGS dataset used in Experiment 1. All words were proper nouns and the categories were self-created (see Appendix C). The category cues that were presented with the word pairs were taken from Freepik (www.freepik.com) and edited to improve the readability of the word pairs. The lists in the control conditions were created using similarity values that were generated in the same way as in Experiment 1.

#### Procedure

Participants were instructed to try to recall the target (in the cued-recall task) and the letters in order of presentation (in the letter span task) as fast and accurately as possible. Participants were told that their bonus depended on the percentage of correct trials (but not the speed at which they responded). Participants were also told that the semantic category cues were meaningful and would be presented again during recall. In total, each participant completed four blocks. Each block consisted of the same three phases as in Experiment 2, but all lists consisted of 20 word pairs. As in Experiment 2, we queried all words in a random order in the recall phase.

To measure the effects of category size on retrieval times and recall accuracy, we varied the number of word pairs associated with each category. In total, we used seven different categories (furniture, sports, colors, vegetables, animals, jobs, and countries). The category cues (i.e., the background pictures) were related to these categories. Pairs were randomly created for each participant, but cue and target were always from the same category. Each participant carried out three blocks. Each block contained two of these categories (unless category size was 20; then, all words in the block were from the same category). To ensure that we had enough data from the small category sizes, two category size combinations where shown for all participants (2–18 and 4–16). To ensure that the rest of the space was also covered, the category size of the third block was randomly sampled from the following options: 0–20, 1–19, 6–14, 9–11. For each participant, we randomly assigned category labels to category sizes without repetitions. In a fourth block, the control block, participants received no category cues and were presented with one of two control lists: In the high similarity pairs control condition, the similarity of each pair was at least 0.7 and the similarity of both the target and the cue to all other words in the list was below 0.3. In the low similarity control condition, all words had a similarity below 0.3 to all other words in the list. In this condition the word pairs were assigned randomly. The order of the blocks was randomized across participants. The rest of the procedure, i.e., presentation times and the three phases (learning, distraction, and recall), were the same as in Experiments 1 and 2.

#### Hypotheses

Building on the predictions of the SimSS model, we had the following hypotheses:

For RTs:

1. The more word pairs associated with a context cue, the slower the RTs for correct responses (see Fig. 2G). As described in Experiment 2 we also wanted to explore potential detrimental effects of larger contexts. 2. Exploratory: Are the RTs for larger context sizes slower than the RTs when there is no context cue controlling for set size? 2. a) Unspecific context cues are detrimental: RTs for larger context sizes are slower than RTs when no context cue is present. 2. b) Context cues are always helpful: the smaller the context size, the larger the benefit for the RTs. If the context size is the same as the set size there is no benefit or disadvantage. 2. c) Unspecific context cues are detrimental, but we can ignore them if they are: a) is true, but if the context size becomes too large, people do not use it as a cue and thus the RTs in the no-context condition represent an upper limit. 3. Retrieval is faster in the control condition, in which cue and target are more similar to each other (see Fig. 2G).

The hypotheses for accuracy were the same as for RTs, but reverse coded (i.e., slower RTs relate to lower accuracy, see Fig. 2 H).

Again, all hypotheses were preregistered (https://osf.io/7ejhc).

#### Analysis

We excluded (a) people who did not provide at least 3 correct responses below 20 s for context sizes below 5 to have enough data per participant for the hierarchical models. And (b) all responses given after 20 s. For the RT analysis, we additionally excluded all incorrect responses. These exclusion criteria were again preregistered.

We tested the hypotheses with regards to RTs using a hierarchical Bayesian linear regression with a lognormal likelihood function implemented in the brms package. To test hypothesis 1 for the RTs, we excluded the data from the control conditions and used the following model:

1. RT $\sim$ context size + (1 + context size | participant)

In addition to analyzing context size as a continuous variable, we also categorized context size into three discrete levels to compensate for the fact that small contexts could only produce few responses (i.e., for context size of 2, we could at most get two responses from each participant). We binned as follows: small context, 1–4; medium context, 5–15; large context, 16–20. To compare the control conditions for the third hypothesis, we analyzed correct RTs from the control conditions using the following model: RT $\sim$ Condition + (1 | participant).

For the accuracy analysis of hypothesis 1, we used the following model:

1. Accuracy $\sim$ context size + (1 + context size | participant)

Same as for RT, we did this analysis with binned and unbinned context sizes. To compare the control conditions for the third hypothesis, we analyzed responses from the control condition using the following model: Accuracy $\sim$ Condition + (1 | participant).

All models were preregistered.

**Fig. 6:**
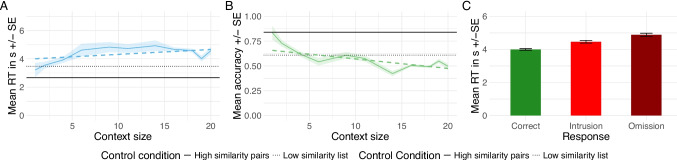
Results Experiment 3. **A** The effect of semantic context on RTs. The two black lines show the mean RTs of the two control conditions. **B** The effect of semantic context on accuracy. The two black lines show the mean accuracy of the two control conditions. **C** The mean RT for the different response types. All shaded areas around the mean and the error bars represent the standard error (SE) of the mean

**Table 2:** Pair-wise comparisons of recall with context and without context

	*RT*		*Accuracy*	
Comparison	Mean difference (in s)	Bayes factor	Mean difference	Bayes factor
Small and high pair similarity	0.9514	$1.46 \times e^{2}*$	$-$0.1540	$4.29 \times e^{2}*$
Medium and high pair similarity	2.1058	$24.6*$	$-$0.4056	$2.4 \times e^{4}*$
Large and high pair similarity	1.8628	$1.77 \times e^{5}*$	$-$0.3403	$9.98 \times e^{8}*$
Small and low list similarity	0.3049	0.38	0.0297	0.24
Medium and low list similarity	0.7611	$11.14*$	$-$0.0428	0.37
Large and low list similarity	0.5395	$3.33*$	$-$0.1018	$1.49 \times e^{2}*$

This shows the results of Bayesian paired-sample *t*-tests using the mean values per participant for the trials with the context sizes and the control conditions. The mean differences were calculated by subtracting the values from the control conditions from the values from the context conditions. Asterisk (*) signifies BF values above 3, i.e., substantial evidence for the alternative hypothesis

### Results Experiment 3

In line with hypothesis 1, RTs for correct responses took longer the more word pairs were associated with a context cue ($\hat{\beta }=0.03$, 95% HDI = [0.01, 0.05], $BF_{10}$ = 1.22, see Fig. 6A). However, the evidence for the effect was only weak when analyzed unbinned, which might be due to the small number of small context responses, or because the effect is non-linear. The evidence for the effect was strong, however, when binning context size into small, medium, and large context bins and analyzing them as factors ($BF_{10}$ = 18.89 for the main effect of context), with medium ($\hat{\beta }=0.15$, 95% HDI = [0.05, 0.24]) and large ($\hat{\beta }=0.11$, 95% HDI = [0.05, 0.17]) context sizes both leading to slower RTs than small context sizes. However, the effect leveled off at larger context sizes, with even a slight decrease in RTs when moving from medium to large contexts ($BF_{10}$ = 4.11). On average, responses for small context sizes were 1.04 s faster and responses for large context sizes where 0.25 s faster than for medium context sizes, respectively. The same trend held for accuracy. Larger contexts were associated with a lower accuracy ($\hat{\beta }=-0.27$, 95% HDI = [$-0.35$, $-0.19$], $BF_{10} > 100$, see Fig. 6B), and binning context size confirmed this effect ($BF_{10} > 100$), with medium ($\hat{\beta }=-0.68$, 95% HDI = [$-1.05$, $-0.31$]) and larger contexts ($\hat{\beta }=-0.85$, 95% HDI = [$-1.08$, $-0.6$]) both having lower accuracy than small context sizes, and larger context having a lower accuracy them medium sized contexts ($BF_{10}$ = 5.09). On average, participants were 14.3% more accurate in smaller contexts than in medium sized contexts and 1.7% more accurate in medium sized contexts than larger contexts. Together, these results suggest that there is a benefit of small context cues. We compared the experimental conditions with a semantic context to the two control conditions without context to investigate whether the presence of a semantic context is actually useful. First, we compared the control conditions to each other (Hypothesis 3). We found that both RTs were on average 0.8 s slower ($\hat{\beta }=0.38$, 95% HDI = [0.14, 0.62], $BF_{10}$ = 36.48) and accuracy was on average 23.2% lower ($\hat{\beta }=-1.47$, 95% HDI = [$-2.06$, $-0.9$], $BF_{10} > 100$) in the low similarity control condition than in the high similarity control condition. This supports the idea that an increased similarity with the target and a decreased similarity with the distractors (i.e., all learned words that are not the target or the cue word) is beneficial for retrieval. To test the benefit of the context size when compared with these control conditions, we compared RTs and accuracy from the binned context sizes with the control conditions using Bayesian paired-samples *t*-tests, using the mean RTs for each participant in the different conditions/bins. RTs were slower and accuracy was lower for larger context sizes when compared to the low similarity control condition. For RTs, this was already the case for medium-sized contexts. For all context sizes, RTs were slower and accuracy was worse when compared to the high pair similarity control condition (see Table 2 for all results).

To see whether this decrease in performance of larger contexts was due to the increasing number of similar none target items (distractors) in the larger context conditions, we used Bayesian paired-sample *t*-tests to test whether the proportion of intrusions increases in the large context condition when compared to the low similarity control lists. We found that there were on average 12.39% more intrusions in the larger contexts ($BF_{10} > 100$). The proportion of omission was roughly the same ($BF_{10}$ = 0.038), supporting the idea, that the decreasing performance is due to the retrieval of more similar distractors.

To further test whether the presence of semantic context cues was detrimental, we also compared Experiment 1 to Experiment 3 (see Appendix B). While the RTs in Experiment 3 was comparable to the performance for the same set size in Experiment 1, accuracy was worse even when only considering the small context sizes.

Post hoc, we also wanted to test if the pair-wise semantic similarity between cue and target still plays a role, even if the cue and target always belong to the same semantic category. To test this, we added a main effect of pair-wise similarity and an interaction between context size and pair-wise similarity for both RTs and accuracy. To check that variability in pair-wise similarities within a category are still present, we plotted the distribution of pair-wise similarities. This plot shows that there is still a large variance of semantic similarities even within a category (see Fig. 12C). For both RTs and accuracy, we found that there was no main effect of pair-wise cue target similarity, and this effect did not change over set sizes (see Fig. 12A and B for a summary of the results). We excluded the data from the control conditions from this analysis and only used the data from correct responses for the RT analysis.

To see whether we can replicate the results from Experiment 1 and 2 that correct responses are faster, we conducted post hoc tests. We found that both intrusions ($BF_{10} > 100$) and omissions ($BF_{10} > 100$) were slower than correct responses with correct responses being 0.71 s faster than intrusions. However, intrusions were only 0.56 s faster than omissions ($BF_{10}$ = 0.19, see Fig. 6C). Accordingly, the trend was the same as in the previous experiments, but we could not show a clear difference between the speed of intrusion and omission responses.

### Discussion Experiment 3

In this experiment, we tested whether semantic context cues produce context effects in LTM retrieval. Our findings show that as category size increased, retrieval accuracy decreased and RTs became slower. This indicates that semantic context cues affect retrieval, and this effect is modulated by the specificity of the context. Notably, this effect was clearer for accuracy than for RTs. And while this might be due to increased noise in the RTs, another plausible explanation is that there is a trade-off between accuracies and RTs. It is imaginable that there is a maximum retrieval time that participants are willing to spend and once this is exceeded a response will be given, even if the correct item has not yet been retrieved. This would result in continuously decreasing accuracies, while RTs only increase to a certain degree.

The use of semantic categories as a context raises the question if the context effects we find are mainly driven by the presence of an increased number of items with a high similarity to the cue. In Experiment 1, we found that an increased similarity between cue and target resulted in an improved retrieval, highlighting the role of continuous semantic similarity. In Experiment 3, despite considerable variability in similarity even within categories, we did not find such an effect, suggesting that participants may have relied more on the semantic context (i.e., the category labels) themselves, instead of the semantic similarity between the cue and potential targets to retrieve items from memory. In other words, the category label itself might be used as the cue (plus some learned association between the current cue and the target). However, given the current evidence, we cannot say for sure if this is the case. Future studies might look at this distinction between the similarity to the cue and the direct use of semantic category labels as cues more closely. Nevertheless, in this manipulation, any effects of context are driven by semantic relatedness in some way.

It is also noteworthy that we did not observe similar context effects in Experiment 2, which suggests that the nature and task-relevancy of context cues are critical. Semantic cues are arguably more directly task-relevant. Unlike visual changes, semantic cues not only enhance cue-target similarity but also provide explicit information about the target (e.g., the target word belongs to the category “animals”).

When comparing performance to the control conditions, we found that these additional cues were often more detrimental than helpful. For medium and large category sizes, accuracy was worse and RTs were slower compared to a list of unrelated words. This highlights the importance of cue specificity: the best retrieval performance occurred when the cue was highly similar to the target while maintaining low similarity to the other words in the list.

Furthermore, performance for larger semantic contexts was worse than for larger set sizes in Experiment 1. This decrease in accuracy for larger contexts was mostly due to an increasing number of intrusions. This fact suggests that retrieving information from larger semantic contexts is particularly challenging and good distractors (i.e., semantically similar items) interfere with retrieval more than unrelated information. The fact that a high density of similar items slows retrieval, even when a cue that is similar to the target is available, supports the idea that memory search is local within a semantic network, meaning that only items that would be in the close vicinity of a cue, but not items that are dissimilar and as such far away will be considered during retrieval. However, the comparison with Experiment 1 should be considered carefully, as we could not control for factors such as lag or the total number of learned items between the two experiments.

**Table 3:** Fitted model parameters

	*Fitted parameters*								
*Model*	$o$	$i$	$a$	$\beta$	$g$	$r$	$\lambda$	Intercept	Stepsize
SimSS	0.015	0.019	0.296	0.429	**0**	**1**	−	600	300
SimSS no Sim	0.015	0.019	0.999	**0**	**0**	**1**	−	600	300
SimSS no AS	0.015	0.019	**0**	0.518	**0**	**1**	−	600	300
SimSS no Sim/AS	0.015	0.019	**0**	**0**	**0**	**1**	−	600	100
SimSS out-of-list distractors	0.015	0.017	0.242	0.405	0.004	**1**	−	400	300
SimSS target rejection	0.014	0.014	0.212	0.482	**0**	0.9998	−	400	300
SimSS softmax	0.015	0.019	0.377	−	**0**	**1**	1.309	600	300

The fitted parameter values for all models. All bold parameters are not fitted, but are set based on modeling assumptions. Empty parameter values do not apply for the corresponding model. Intercept and stepsize are in milliseconds. Everything is rounded to the 3rd decimal, except for $r$, to highlight that this value is close, but not equal to 1

Ultimately, these findings demonstrate that semantic context manipulations are more likely to influence memory retrieval than unrelated visual changes. This distinction aligns with real-world scenarios, in which context is often semantically meaningful. For example, it is more likely that I will need to remember a recipe in the kitchen than in the bathroom.

## Model Results

As we showed in the introduction of the SimSS model, a sequential sampling model can qualitatively capture the effects of set size and semantic similarity that we observed in Experiment 1, as well as the effects of semantic category size observed in Experiment 3, on both accuracy and RT. We now sought to test whether the SimSS model could also provide a good quantitative fit to the data, and whether the mechanisms we introduced (semantic similarity and association strength) are necessary to achieve this fit. Additionally, we examined whether further assumptions or model extensions could improve its performance.

To fit the model, we used maximum log-likelihood estimation based on a joint measure of response type and reaction time. For details on the fitting, see Appendix C. Based on the above results, we fitted the models only using the data we included in the analysis for Experiments 1 and 3.

To test our main assumptions, as well as some extensions, we fitted the following models:

1. SimSS: The full SimSS model as described above.
2. SimSS no Sim: To test whether similarity plays an essential role to fit the data quantitatively, we implemented a model that does not take the semantic similarity into account. We operationalized this by setting $\beta = 0$. This effectively means that each word has the same probability of being sampled (with an increased probability for the target due to the added association strength). As such this model has only three parameters ($i, o \text { and } a$).
3. SimSS no AS: To test whether the association strength, i.e., the association between cue and target beyond the given semantic similarity, is necessary, we implemented a model without this association. As such this model has only three parameters ($i, o \text { and } \beta$).
4. SimSS no Sim/AS: The SimSS model without association strength and similarity. This model serves as a baseline. As such, this model has only two parameters ($i, \text { and } o$).
5. SimSS with out-of-list distractors: This model includes all words from previously encountered lists. However, the similarity of these words is scaled by an out-of-context devaluation parameter $g$. This allows us to directly test the influence of words from other lists on the retrieval process, both by comparing this model to the orginal SimSS model and by looking at the value of $g$. If $g$ is close to zero, this would indicate that out-of-context words have little to no influence on the retrieval process. As such, this model now has five parameters ($i, o, \beta , a \text { and } g$).
6. SimSS with target rejection: To test whether it is a valid assumption that if a target gets sampled, it will always be accepted (i.e., retrieved correctly), we fitted a model with an added acceptance parameter $r$. If $r$ is close to 1, this would justify our simplification for the original model. As such, this model now has 5 parameters ($i, o, \beta , a \text { and } r$).
7. SimSS with softmax: Here, we use the softmax function to calculate the sampling probabilities based on $$s(k,c)$$. This allows us to investigate whether the similarity space needs to be transformed to better reflect the retrieval process we observed. Many transformations are possible. We used the softmax as one possible example. This model still has four parameters ($$i, o, a $$, and instead of $$\beta $$, it has a $$\lambda $$ parameter for the softmax function). The larger $$\lambda $$ is, the more likely only items with high similarity values will be sampled.

We additionally fitted the stepsize parameter of the geometric distribution and an intercept parameter (i.e., a constant intercept on RTs related to encoding the cue and executing the response) via a rough grid search to test what values are a good account of the empirical data (see Appendix C for details). These parameters were not included in the BIC calculation, because they were common across all models. The parameter values of the fitted versions of these models are summarized in Table 3.

**Fig. 7:**
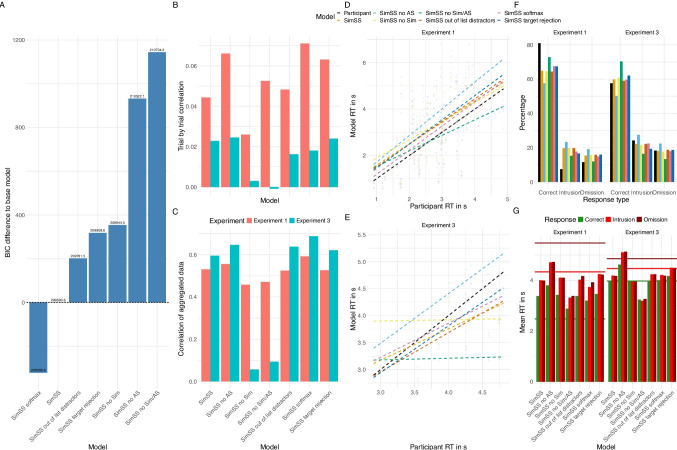
Model fitting results. All plotted model results are based on 10 simulations across all trails we included in the analysis of experiment 1 and 3 using the fitted parameters of each model. The “Participant” model refers to the actual data. **A** The BIC difference to the full SimSS model as described in the model predictions section. Lower values represent a better fit. **B** The trial by trial correlation between the RTs generated by the models and the actual RTs that participants produced. **C** The correlation of the same simulations as in **B**. However, here the RTs are aggregated across the experimental conditions (set size, cue target similarity (binned into 5 bins), context size and the control conditions of experiment 3). **D** and **E** The aggregated data points from **C** and a linear regression of these aggregated data for each model for Experiments 1 and 3, respectively. We also added the actual data (in black) as a reference. **F** The percentage of each response type across the 10 simulations of each model compared to the actual percentage (in black). The model colors are the same as in **D** and **E**. **G** The mean RT generated by each model for each response type. The solid lines are the corresponding Participant RTs

We used the Bayesian information criterion (BIC) to compare the different models. The BIC is a model comparison metric that measures the goodness of fit while penalizing for model complexity, helping to identify the most parsimonious model that explains the data. Smaller BIC values indicate a better fit. We observed that the best model was the model with the softmax transformation of the similarities (see Fig. 7 A). None of the other models was able to explain the data better than the base SimSS model. To better understand how the predictions diverge from the actual data, we simulated each model 10 times across the trials of Experiment 1 and 3 (only trials included in the analysis) with the fitted parameters. The model results and these simulations indicate the following conclusions when considering cued recall in proposed sequential search framework. (1) Correlating model predictions with trial-level data reveals substantial residual variance (see Fig. 7B). However, the variance produced by our experimental manipulations is relatively well-explained (see Fig. 7C, D, and E) by at least the full version of the model. This difference highlights that the SimSS model is a very simple minimalist model, which ignores a lot of sources as variance, some of which would be hard to model at all (such as random variances in the motor response), while others might be explainable (such as variance that might be caused by the fidelity of the encoding process), but are not the focus of this study. (2) Similarity emerges as a critical factor in explaining recall behavior. Models excluding similarity not only had higher BIC scores but also failed to capture key data patterns, especially in Experiment 3 (see Fig. 7C and E), in which differences in similarities due to the semantic categories were central to the design. (3) The model without the association strength can capture the RT pattern well, but seems to overestimate the RTs (light blue in Fig. 7D and E). Moreover, and consistent with the role of association strength as a proxy for the learned word-pair association, this model failed to reach participant-level accuracy (see Fig. 7F). (4) While most models correctly reproduce the general pattern that correct responses are faster than errors, they systematically underestimate the RT difference. Additionally, none of the models fully captured the observed pattern that omissions tend to have longer RTs than intrusions (see Fig. 7G). This highlights one of the shortcomings of this model and shows that this model needs further extensions to capture both RT and accuracy data. (5) The results also suggest that certain simplifications may be justified. The target acceptance parameter $r$, introduced to model a recognition-like gate for correct targets, was estimated near 1 and did not improve model fit, indicating that omitting an explicit recognition stage may be a valid assumption in this context. (6) Similarly, the devaluation parameter $g$ for out-of-list distractors was close to zero, implying that these items do not substantially contribute to the observed effects. This raises an important theoretical question about the mechanisms by which memory search can restrict itself to the relevant list or context, an issue we return to in the general discussion.

In sum, the modeling results support the view that a similarity-based sequential sampling process, coupled with learned association strength, provides a useful explanatory framework for cued recall behavior. The SimSS model, despite its simplicity, captures key patterns in the data when both components are present, including the effects of experimental manipulations and the relative timing and accuracy of responses. While the SimSS model does not capture all trial-level variability or RT patterns for different error types, improvements can likely be made with some targeted alterations. Here, we presented one such alteration in the form of the transformation of the semantic similarities via the softmax function. This already led to a better model fit.

## General Discussion

In a series of three experiments using a cued-recall task, we investigated how retrieval from LTM is influenced by the set size of a to be learned list and the similarity between cue and target. We tested whether additional visual and semantic context cues help us retrieve a target faster, and whether successful retrieval depends on the specificity of these cues. In Experiment 1, we demonstrated that increasing the number of to be learned word pairs leads to slower RTs and lower retrieval accuracy. Furthermore, the semantic similarity between the retrieval cue and the target word facilitated retrieval, regardless of set size. The set size effects depended on the list context. Specifically, we observed set size effects only within, but not across lists, indicating that participants can successfully use a list context to constrain the set they search. In Experiment 2, we explored whether visual context cues can similarly be used to constrain memory retrieval but found no effect of context size on RTs. But we could show in Experiment 3 that larger semantic contexts were associated with slower RTs and lower accuracy and led to worse recall when compared to lists of unrelated words. Furthermore, we also showed that similarity measures based on vector embeddings learned from text corpora are a useful approximation of human similarity ratings that can explain some behavioral phenomena.

First, we consistently observed that recall performance decreased when an increasing number of word pairs had to be remembered. In doing so, we support the findings from Nobel and Shiffrin (2001) who reported a similar set size effect for cued recall. However, while these finding seem intuitive, they and similar findings, particularly in recognition, have recently been called into question by Kinnell and Dennis (2011), who showed that there is no set size effect in recognition memory when controlling for confounding variables such as the retention interval (lag), attention, displaced rehearsal and contextual reinstatement. Despite controlling for these factors in Experiment 1, we still observed the set size effect in a cued-recall paradigm. Furthermore, everyday experience suggests that we somehow do cope with far larger set sizes without unreasonably long retrieval times, which is supported by findings in recognition memory that suggests that even with set sizes that are two magnitudes larger then what we used here, item recognition accuracy was still close to ceiling (Brady et al., 2008). The fact that this does not hold for cued-recall in our experiment to the same degree raises the question of why this is, especially since we found that both RTs and accuracy were affected. If this effect continued for larger set sizes, then at some point, people would not be able to recall anything. This is especially true if we assume a trade-off between accuracy and response times, whereby individuals terminate their memory search after a certain duration. Here, we argued that this divide is due to the way memory is organized and the qualities of the cue, meaning that we are affected by set size, but we are affected locally. In other words, not the total number of memories but the number of memories related to a memory cue is what negatively affects memory retrieval. Strategies that allow us to increase and decrease the similarity between target memories and potential distractors are therefore essential.

We look at memory retrieval as a search process through stored representations. To enable quick retrieval, despite an increasing number of memories, we endorse the idea that memory is organized according to the similarity between memory representations, such that two similar representations are stored closer in this space than two dissimilar representations (Kumar, 2021). Accordingly, we assume that the similarity between cue and candidate items (i.e., the distance in the organized space) guides the search.

One such organizational feature is semantic similarity. The fact that target words were more easily recalled when the cue was semantically similar to the target suggests that semantically similar items are more closely connected in the memory network and can be accessed more efficiently, supporting theories of memory organization based on semantic similarity (Kumar, 2021; Shiffrin & Atkinson, 1969). Alternatively, one might argue that improved RTs and accuracy for more similar words might be caused by the relative advantage of more similar items during encoding rather than retrieval. This effect is also known as the list strength effect, which refers to the observation that memory for strongly encoded items is harmed by the presence of other strongly encoded items and helped by the presence of other weakly encoded items (Wixted et al., 1997). Here, it could be that more semantically similar items are simply encoded more strongly as compared to semantically less similar items. And the presence of these less strongly encoded items helps the recall for the more strongly encoded items. However, then, we should not have observed improved performance for the high similarity pairs condition in Experiment 3, since here all items should be strongly encoded and according to the list strength effect that should harm the recall of all items. This supports the lack of a list strength effect in cued-recall (Wilson & Criss, 2017) and the idea that semantic similarity aids memory retrieval, independent of encoding strength.

Context too, in this view of similarity based retrieval, could have a large influence on the similarity between the cue and potential targets and could thus influence retrieval accuracy and time. Here, we looked at three types of context: temporal (list membership), semantic (category membership), and visual (background scenes), and asked whether they exert similar or distinct effects on memory performance and whether set size effects can be explained by the context size.

In Experiment 1, we observed that the temporal context of a study list effectively segmented memory: increasing the number of items within the current list impaired retrieval, yet items from other lists, despite the close succession in which they were studied, had no measurable impact on performance. This suggests that temporal boundaries serve as strong constraints, allowing retrieval to be constrained to items within the current context. This idea is supported by prior work emphasizing the crucial role of temporal context in memory organization (Brown et al., 2007; Jang & Huber, 2008; Farrell, 2012). Temporal context can, however, also hinder retrieval, as the increasing number of items within a list still negatively impacted retrieval speed and accuracy. This is consistent with past findings that show that temporal proximity leads to retrieval errors when contexts are less distinct (e.g. Henson, 1996; Hurlstone & Hitch, 2015; Lee & Estes, 1977; Nairne, 1991; Smyth et al., 2005). Thus, temporal context can both aid and hinder retrieval, depending on its specificity (i.e. how many items are associated with a context). The modeling results further support the conclusion that temporal context can constrain memory search by showing that including out-of-list items in the search did not further improve model fit.

Semantic context, in contrast, did not provide such precise segmentation. In Experiment 3, increasing the number of semantically related items impaired memory performance, indicating that these items competed during retrieval. Crucially, even small semantic contexts did not boost performance to levels observed in smaller lists in Experiment 1. Instead, even for just medium sized contexts, performance was worse when compared to a list of unrelated word pairs without context cues.

These findings suggest that semantic contexts might not function as retrieval contexts in the same way that temporal contexts do. Indeed, unlike semantic categories in our task, which were queried together with items from a different semantic context in the same list, temporal contexts defined the exclusive set of potential targets. This asymmetry in relevance may have given temporal context a stronger functional role in the retrieval process. Another possibility is that semantic and temporal contexts differ only in degree, not in kind: perhaps temporal context more strongly increases cue-target similarity or is more tightly bound to the encoding episode. Or semantic similarity might have such a strong effect that even if the memory search is constrained to only items from the same category, these items act as strong distractors (lures), more so than a set of unrelated items. This could result in worse performance even for smaller contexts (this assumption could be tested in future studies by having lists that vary in size, but only contain words from one semantic category and compare them to lists with several semantic categories that vary in the category size, not the list size). With our current result, we cannot definitively say which of the two explanations is more likely.

The lack of an effect of visual context in Experiment 2 contrasts with the observations in Experiments 1 and 3. The null effect is consistent with previous results on visual context, which have been inconclusive. Some studies have demonstrated the effectiveness of visual context cues (Liu et al., 2024; Verkoeijen et al., 2004) while others have reported null effects. For example, the often cited diver study (Godden & Baddeley, 1975), which varied not only the visual, but also the proprioceptive context, could not be replicated (Murre, 2021). One possibility is that visual background features are processed differently when encoding words and are less relevant for cueing retrieval. However, we favor a more pragmatic explanation: visual context may fail to impact retrieval unless it is attended to or perceived as relevant. Participants may have suppressed the visual backgrounds due to their irrelevance for the task, consistent with findings that visual or environmental context effects are diminished when attention is directed elsewhere (Smith & Vela, 2001; Logan & Etherton, 1994). Thus, the role of visual context may not be fundamentally different but contingent on encoding depth and attentional engagement. Overall, the results remain inconclusive, and the factors determining when visual context is effective warrant further investigation.

If different contexts can be treated under a unified framework, for example, as features along a shared similarity dimension, then a key goal for future research will be to map the dimensions of different context features onto this common space and determine how attentional and task-driven factors modulate their contribution to retrieval.

In the beginning of the paper, we proposed that the usefulness of the additional cues a context provides depends on two factors: (1) How much the context increases the similarity between cue and target. (2) How distinctly the context is associated with the target. Irrespective of whether different context features act functionally different, we show that an increased similarity between context and cue through contextual features improves recall. We also found that the helpfulness of a context depends on the specificity. However, our findings also suggest that additional context cues that are even slightly unspecific are detrimental rather than helpful. This would mean that having any number of highly related memories would make the recall of any of them more difficult.

Also noteworthy, we show that a similarity measure based on Word2Vec vector embeddings can be useful to explain human behavior. Specifically, we found that this measure is correlated with pair-wise ratings, a common method to ask people about the similarity between things (Rubenstein & Goodenough, 1965). We also found that they can be used to explain and model search times and accuracy in LTM. Other research using a host of different models also showed that these embeddings from models trained on text corpora capture some of the human intuition of what it means to be similar (Marjieh et al., 2022; Richie & Bhatia, 2021). This highlights the usefulness of these embeddings in computational modeling of behavior, especially when considering the easy accessibility of these embeddings, when compared to human similarity ratings.

An alternative explanation for the observed set size and similarity effects is that they arise from limitations, such as divided attention or encoding interference, during encoding rather than retrieval. Specifically, as set size increases, attentional resources may be more thinly distributed across items, leading to weaker or less distinct memory traces. Similarly, higher similarity among items could result in greater interference during encoding, increasing the likelihood of overlapping representations. Ruling out the influence of encoding related differences is hard; however, we tried to mitigate such effects by implementing a strong encoding phase across conditions, including practice opportunities. Furthermore, the use a proactive design to control for differences in temporal distance between study and test should rule out any effect due to the degradation of memory traces. Additionally, if encoding quality declined with set size due to divided attention, we would expect systematic differences between blocks as the attention decreased throughout the experiment. The lack of such effects suggests that the attention did not vary much throughout the experiment. In conclusion, while we can not fully rule out encoding limitations as a contributing factor, the absence of such markers and the structure of our design support a more parsimonious explanation in terms of retrieval interference and sequential search.

The proposed similarity-based sequential sampling (SimSS) model offers a framework for understanding memory retrieval as a search process driven by (semantic) similarity. Building on theories such as ARC-REM (Diller et al., 2001) and SAM (Raaijmakers & Shiffrin, 1981, 1980), SimSS posits a high-dimensional memory space in which retrieval cues guide a probabilistic search for target items. Our results demonstrate that predictions by this sequential mechanism, which samples items based on their similarity to the cue and association strength between cue and target, align with key behavioral patterns observed in our experiments, such as the set size effect, the improvement through cue–target similarity, the increased RTs for incorrect responses, the context effects for semantic categories, and the advantage of target-specific cues in memory retrieval.

To test which features of the model are necessary to explain the observed data, we compared several versions of the SimSS model. This model comparison confirmed that both the semantic similarity and the association strength are necessary to capture the observed effects. Models without similarity failed, for example, to explain the patterns observed in Experiment 3, in which semantic categories played a crucial role. Similarly, models without an association strength parameter did not manage to account for the accuracy that participants manage to achieve. These results support the notion of a search based on semantic similarity and the necessity of strengthening the cue target association.

We implemented the SimSS model as a minimalist model, which tries to model cued recall with as few parameters as possible. In doing so, we made several simplifications, such as removing a detailed recognition process for correct items and only including items from the current list in the model. To test whether these simplifications are justified, we compared the base model to versions without these simplifications. However, adding a recognition parameter or out-of-list distractors did not substantially improve the fit, with the fitted parameters indicating that recognition was nearly perfect and that the inclusion of out-of-list distractors does not help in explaining our results. However, the fact that we do observe out-of-list intrusions does indicate that this implementation is too simplistic. Likely, the list context does not present an absolute boundary. However, given the few intrusions we observe, here, we have no way of investigating how and when and out-of-list items are retrieved or considered for retrieval.

We also wanted to investigate whether semantic similarities are weighted linearly. By introducing a scaling parameter (i.e., $\beta$) for the semantic similarity values of the SimSS model, we already tested this and showed that smaller similarity values might be weighted more strongly. However, a different transformation might even better capture the effect of similarity on memory retrieval. And indeed, we found that applying a softmax transformation to the similarity space improved the model fit, indicating that items with larger similarities are disproportionally more likely to be sampled.

Overall, despite its minimal assumptions, the SimSS model captures the main effects of the data well, supporting the ideas that (1) retrieval is guided by similarity, (2) memory search is a sequential process, and (3) activation or sampling probability scales with set size. As we noted in the alternative model sections, other models of retrieval can also account for these results. But often they are more complex or would need additional assumptions to model RTs. By showing that only few assumptions are needed to account for a lot of the effects we observed in cued recall, we hope to provide a simple lens through which we can view the retrieval of specific memories given a cue.

Nevertheless, some discrepancies between model predictions and empirical data highlight areas for future improvement. For example, while the fitted SimSS model seems to capture the aggregated data (across the effects of interest here) well, a lot of variance in the raw data remains unexplained. Furthermore, unlike the data, the SimSS model predicts a set size effect on RTs for incorrect responses, which was not observed. Similarly, the model fails to capture the pattern that omissions are slower than intrusions. These limitations indicate that additional mechanisms are needed to capture memory retrieval more fully. For example, while SimSS assumes that correct items are immediately recognized upon sampling, a simplification justified by the near-perfect recognition parameter in our fits, future work could explore more detailed models of recognition processes, as suggested by earlier frameworks such as ARC-REM (Diller et al., 2001). Furthermore, at the moment, the context dimensions of time and semantic similarity are not implemented in the same way. This could be improved in our model or similar models to create a more complete understanding of memory retrieval.

One other important limitation of the presented framework is that the SimSS model is only a model of retrieval and we ignored all encoding processes. Despite this, we do believe that there were effects of encoding on the recall performance in our study. However, we tried to minimize any systematic effects of encoding by emphasizing the encoding phase, allowing us to focus on how memories are accessed without modeling how they were formed. Nevertheless, to create a full account of memory, encoding dynamics will have to be incorporated.

Finally, while SimSS was developed for cued recall, future research could test its applicability to other retrieval tasks. For example, for free recall, SimSS would not predict any set size effects for RTs, since the sampling time of each item is constant and any found item is a correct item. Testing such predictions could provide insights into the generalizability and limits of sequential sampling accounts of memory retrieval.

## Conclusion

Overall, the results support the notion of a sequential search process in memory that is modulated by both similarity and contextual information. Notably, we not only show the importance of the cue-target similarity, but also the importance of target specific cues. Furthermore, we show the importance of context, but also that different dimensions of context affect retrieval from LTM differently and that not all context features affect retrieval. In doing so, we contribute to the understanding of context and memory organization. Future research should further investigate the contextual dimensions that influence memory retrieval as well as the role of the set size effect in more naturalistic conditions and how it is overcome there.

### Declaration of Generative AI and AI-Assisted Technologies in the Writing Process

During the preparation of this work, the authors used ChatGPT in order to improve the readability of single paragraphs or sentences. After using this tool/service, the authors reviewed and edited the content as needed and take full responsibility for the content of the publication.

## Figures and Tables

**Fig. 8:**
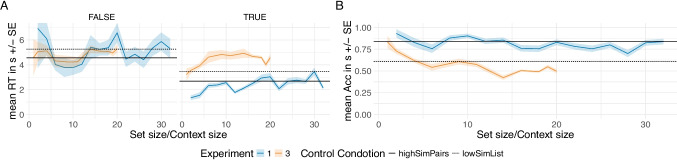
Comparison Experiments 1 and 3. For all plots, the *x*-axis represents the set size for the data from Experiment 1 (blue lines) and the context size for the data from Experiment 3 (orange lines and black lines) to make the effects of the two more comparable. **A** The average RTs for Experiment 1 and 3. The left panel shows the RTs for incorrect responses and the right panel the RTs for correct responses. **B** The average accuracy for the two experiments

**Fig. 9:**
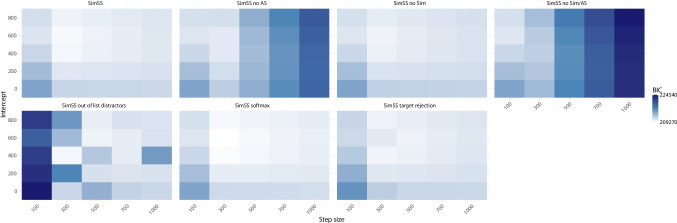
Shown are the BIC values of the fitted models across various stepsizes and intercepts. We used this coarse grid search to determine the best stepsize and intercept for each model

**Fig. 10:**
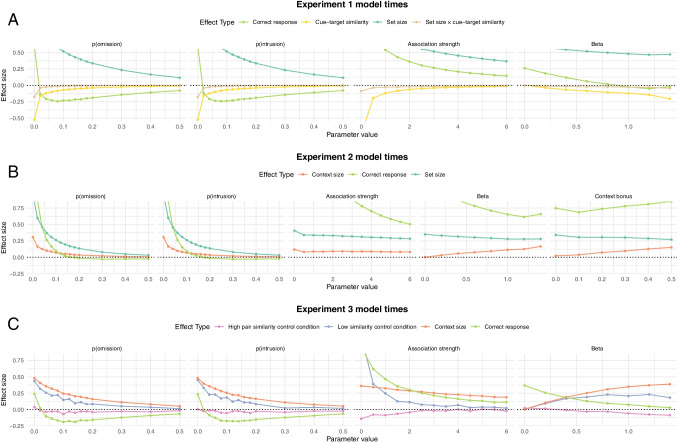
Each panel shows the effect size on model response times across the relevant parameters. The different effects/predictors we looked at are shown in different colors. Negative effects mean a reduced model response time due to that predictor and positive effect sizes mean an increased model response time. We did not include a measure for variance as we can arbitrarily reduce that depending on the number of simulations we run

**Fig. 11:**
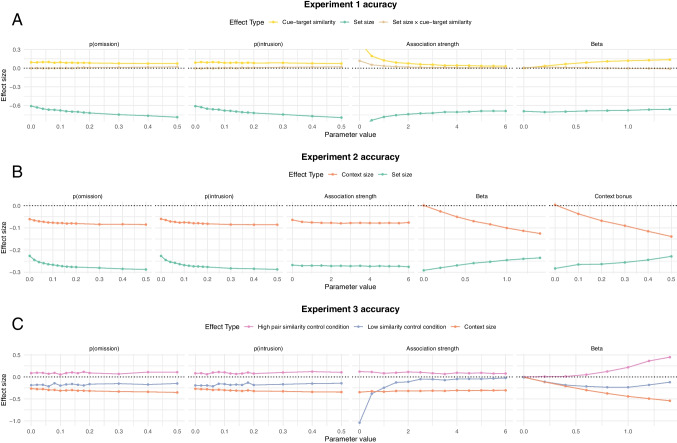
Each panel shows the effect size on accuracy across the relevant parameters. The different effects/predictors we looked at are shown in different colors. Negative effects mean a reduced accuracy due to that predictor and positive effect sizes mean an increased accuracy. We did not include a measure for variance as we can arbitrarily reduce that depending on the number of simulations we run

**Fig. 12:**
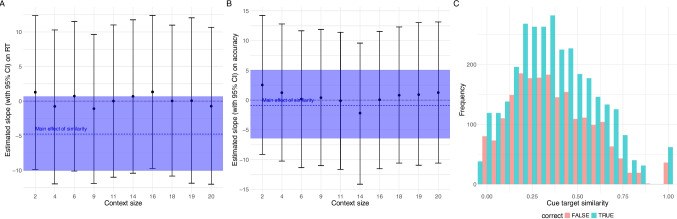
Effect of cue-target similarity in Experiment 3. **A** The slopes of the similarity on RTs of the mixed effects regression for the different context sizes. The main effect of similarity and the 95% CI is plotted in blue. **B** The slopes of the similarity on accuracy of the mixed effects regression for the different context sizes. The main effect of similarity and the 95% CI is plotted in blue. **C** A histogram of the cue-target similarities of Experiment 3 for all word pairs from the conditions where we manipulated the context size. Shown are only the values for the condition with a context size. All error bars represent the 95% CIs

## Data Availability

All code and data have been made publicly available and can be accessed https://github.com/susanneharidi/memoryscaling.

## Appendix A: Similarity Validation

In this separate pilot study, we tested whether the cosine distance between pretrained Word2Vec embeddings is a good account of human pair-wise similarity ratings. To do so, we collected human pair-wise similarity ratings using the word pairs from Experiment 1.

We collected data from 50 adults (age range, 18 to 45) via Prolific. To ensure that the task was understood correctly, all participants had to answer three comprehension questions before the start of the experimental trials. We also had attention checks during the experiment, which 3 participants failed, leaving us with data from 47 individuals. Participants were paid $3.00. Most participants finished the experiment in under 8 min. Informed consent was obtained from all participants before the experiment started. The study was approved by the ethics committee of the medical faculty of the University of Tübingen (number 701/2020BO).

People were instructed to rate 55 word pairs on their subjective semantic similarity on a scale from 0 (not similar) to 100 (very similar). Five of the 55 words pairs were attention checks, where we presented the same word twice. People that did not rate the similarity above 95 for all of these attention checks were excluded from our analysis. The other 50 word pairs were randomly taken from the set of word pairs presented in Experiment 1. In total, we acquired 2350 similarity ratings for 139 word pairs (on average 16.9 (min = 9; max = 25; SD = 3.32) ratings per word pair).

For each word pair, we calculated the average rating, which we used as the human similarity rating. We then correlated that value with the cosine similarity of the same words calculated by the pretrained “en_core_web_md” Word2Vec model.

Human pair-wise similarity ratings and the Word2Vec-based similarity measure had a correlation of 0.81.

Based on these results, we felt confident in using the Word2Vec-based similarity measure for all our analyses. This is very relevant, since the Word2Vec-based measure represents a readily available estimate for similarity, which is far easier to obtain and can easily deal with changing word stimuli. Because of this high correlation, we feel justified in taking advantage of the benefits that the Word2Vec-based measure provides.

## Appendix B: Comparison between Experiments 1 and 3

To better understand potential benefits of additional context cues and whether these cues can overcome the set size effects we observed in Experiment 1, we compared RT and accuracy data from Experiments 1 and 3.

First, we compared the average RT and accuracy of each of the conditions in Experiment 3 with the data from the 20 set size condition in Experiment 1. Since these conditions all had the same set size we used all data from all trials. We excluded trials with RTs above 20 s, and for the RT analysis, we only looked at correct responses. Using an independent Bayesian *t*-test, we found that when compared to Experiment 1, there was no difference in RTs to the context conditions (i.e., the average RT across all context sizes) of Experiment 3 ($$BF_{10}$$ = 0.66), and no difference to the high similarity pairs ($$BF_{10}$$ = 0.92) and low similarity list ($$BF_{10}$$ = 0.25) control conditions. However, participants in Experiment 1 were 29.7% and 21.3$ more accurate in then in the context ($$BF_{10} > 100$$) and low similarity list ($$BF_{10}$$ = 46.94) conditions of Experiment 3. There was no difference when compared to the high similarity pairs condition ($$BF_{10}$$ = 0.26). Even when only comparing the performance of context sizes below 5 with the performance from Experiment 1, participants were still on average 16.1% more accurate ($$BF_{10}$$ = 10.05), even if there was no difference in the RTs ($$BF_{10}$$ = 0.25).

This comparison has to be taken with caution, since we have very limited data from Experiment 1 for this comparison and the similarity manipulation differed between the two experiments. Nevertheless, these results indicate that the additional semantic context cues where detrimental rather than helpful and where not used to overcome set size effects. Rather, the heightened similarity to distractors in the larger contexts in Experiment 3 seems to negatively affect recall accuracy while not reducing RTs. This indicates that either the whole set size still effects recall and the semantic categories cannot be used to constrain the search, or the search can be constrained by semantic category, but the effect of even just a few good distractor items has such detrimental effects, that they compensate for any benefits a reduced considered set size might bring.

## Appendix C: Model Fitting

To fit the model, we used maximum log-likelihood estimation based on a joint measure of response type and reaction time.

The probability of producing a correct response, an omission or an intrusion at any sampling step can be calculated by following the flowchart in Fig. 1A. The probability of producing a correct response in a single sampling step $(p(correct))$, green arrow in Fig. 1A) is as such determined by the following:

4 $$\begin{aligned} p(correct) = p(t_c) = \frac{s(t_c, c)}{\sum _{j=1}^{N} s(j,c)} \end{aligned}$$

with $t_c$ being the target item. The exact implementation of $s()$ depends on the model version with the basic version being the same as described in the introduction of the SimSS model (Eq. 1). Given this probability, we can now determine the probability of an intrusion (light red arrow in Fig. 1A) and an omission (dark red arrow in Fig. 1A) in every given sample step:

5 $$\begin{aligned} p(intrusion)= &  (1-p(correct)) \cdot i \end{aligned}$$

6 $$\begin{aligned} p(omission)= &  (1-p(correct)) \cdot o \end{aligned}$$

However, since these responses only occur if the search has been stopped, we also need the stopping probability to calculate the models probability of a certain response given the observed response. Since stopping only occurs when one of these three responses has been given, the probability that the process stops at every given step is as follows:

7 $$\begin{aligned} p(stop) = p(correct) + (1- p(correct) ) \cdot (o + i) \end{aligned}$$

By using these probabilities, it is straightforward to show that the log-likelihood for the response type given the model parameters is as follows:

8 $$\begin{aligned} \log L_{\text {total}} = \sum _{m=1}^{M} \log \left( \frac{f(\text {R}_m, p(correct)_m,o, i)}{p(stop)_m} \right) \end{aligned}$$

where the function $f(\text {R}_m, p(correct)_m, o, i)$ depends on the observed response $R_m$ in trial *m*:

9 $$\begin{aligned} f({\text {R}_m, p(corret)_m, o, i}) = {\left\{ \begin{array}{ll} p(correct)_m & \text {if R}_m = \text {``C''} \\ (1 - p(correct)_m) \cdot o & \text {if R}_m = \text {``O''} \\ (1 - p(correct)_m) \cdot i & \text {if R}_m = \text {``I''} \end{array}\right. } \end{aligned}$$

with “C,” “O,” and “I” standing for correct, omission, and intrusion responses, respectively.

We then use the number $S_m$ of candidate words sampled in trial *m* to model the reaction times. Specifically, using Eq. (7), it is straightforward to show that $S_m$ has a geometric distribution with parameter $p(stop)_m$. Then, for a given number $S_m$ of samples, we assume that the reaction time $RT_m$ at trial *m* is sampled from a uniform distribution:

10 $$\begin{aligned} RT_m \sim {\left\{ \begin{array}{ll} \text {Uniform}(0, \text { stepsize} +\text {intercept}) & \text {if } S_m = 1 \\ \text {Uniform}(\text {intercept} + (S_m-1) \cdot \text { stepsize},\\ \text {intercept} + S_m \cdot \text { stepsize}) & \text {otherwise} \end{array}\right. } \end{aligned}$$

where stepsize and intercept are free parameters of the model. The stepzise models how long each mental sampling step takes and the intercept accounts for some initial time needed for encoding and responding. Using these assumptions, we can then convert every RT to a discrete number of sampling steps:

11 $$\begin{aligned} S = \max \left( 1,\left\lceil \frac{\text {rt} - \text {intercept}}{\text {stepsize}} \right\rceil \right) - 1 \end{aligned}$$

where $S$ is the number of sample steps and $\lceil$ refers to the next higher integer. Then, the total log-likelihood of reaction times is given by the following:

12 $$\begin{aligned} \log L = \sum _{m=1}^N \left[ \log \left( \text {Geom}(S_m; p(stop)) \right) - \text {adjustment\_term}_m \right] \end{aligned}$$

with the adjustment term being defined as follows:

13 $$\begin{aligned} \text {adjustment\_term}_m = {\left\{ \begin{array}{ll} \log (\text {stepsize} + \text {intercept}) & \text {if } S_m = 0 \\ \log (\text {stepsize}) & \text {otherwise} \end{array}\right. } \end{aligned}$$

This adjustment term is needed to account for the fact that there is a uniform distribution over all RTs that correspond to one sample step (as shown in Eq. (10)). This means that the larger a sample step is, the higher is the uncertainty of a RT given a sample step (and thus the adjustment term).

We used the optim function in R with the implementation of the optimization algorithm proposed by Nelder and Mead (1965). Since the stepsize and the intercept can only be fitted in an outer loop, which is very computationally expensive, we did a coarse grid search over 5 intercept and 5 stepsize values. We present the results for the model with the best BICs over these parameters. The results of the grid search are shown in Fig. 9.

## Appendix D: Robustness Check of the Model Predictions

To ensure that our model predictions were not overly dependent on specific parameter choices, we conducted a robustness analysis. We systematically varied the parameters of the SimSS model within plausible ranges, reran the simulations described above for each combination, and used linear regression to estimate the effect sizes of the predicted outcomes. Specifically, we varied the omissions probability $o$ and the intrusion probability $i$ between 0 and 0.2. We chose this range, as larger values are likely to result in a large amount of errors. The association strength ($a$) was varied between 0 and 6, which is a relatively large range (since semantic similarity $sem(k,c)$ only varies between 0 and 1), but as we did not know how strong the binding between cue and target would be, we wanted to err on the save side here. Finally we varied the scaling parameter for the similarities ($\beta$) between 0 and 1.5. The results are summarized in Fig. 10 for the model response times and Fig. 10 for the accuracy. Overall, the predicted effects reported in the main manuscript remained robust across parameter settings, with a few notable exceptions. In Experiment 1, the interaction between set size and cue–target similarity was only present in the RTs for some parameter combinations. Specifically, the more likely some type of response is given (omission, intrusion, or correct response), the smaller the interaction becomes. This makes sense, because the probability that the search is ended early increases. As expected, higher values of $o$ and $i$ reversed the pattern that correct responses tend to be faster. Additionally, setting $\beta = 0$ eliminated similarity-based effects and the influence of semantic category size in Experiment 3, as expected. All other effects were preserved and varied in predictable ways across the tested parameter space.

## Appendix E: Word Categories of Experiment 3

### Country Words

chile, china, cyprus, egypt, england, ghana, india, italy, japan, jordan, kenya, kuwait, latvia, liberia, malta, mexico, nepal, niger, oman, qatar, russia, samoa, senegal, serbia, sierra, spain, sudan, sweden, syria, togo, tonga, tunisia, uganda, yemen, zambia, angola, germany, bhutan, brazil, canada, congo, fiji, france, gambia, greece, guinea, israel, malawi, norway, poland

#### Job Words

baker, chef, coach, coder, diver, editor, guide, judge, lawyer, miner, model, nurse, doctor, pilot, plumber, poet, porter, rabbi, ranger, scout, smith, priest, tailor, teller, tutor, usher, teacher, valet, mayor, welder, writer, actor, agent, artist, barber, buyer, clerk, dancer, driver, envoy, farmer, guard, janitor, jester, medic, monk, painter, owner, sailor, singer

#### Animal Words

bison, camel, bear, cheetah, cobra, coyote, wolf, dolphin, donkey, eagle, ferret, gecko, gibbon, goose, horse, jaguar, koala, lemur, lion, lizard, llama, macaw, mole, monkey, moose, otter, panda, parrot, pigeon, rabbit, raccoon, salmon, seal, shark, sheep, skunk, sloth, snail, spider, squid, swan, tiger, toad, trout, turtle, viper, frog, weasel, whale, zebra

#### Vegetable Words

arugula, bamboo, beet, carrot, chard, celery, chives, collard, garlic, ginger, kale, kohlrabi, leek, lettuce, okra, onion, parsnip, chili, pepper, radish, rhubarb, eggplant, shallot, spinach, squash, tomato, turnip, wasabi, zucchini, broccoli, cabbage, endive, chicory, fennel, celeriac, basil, potato, lentil, daikon, oregano, cilantro, parsley, thyme, rosemary, ramp, taro, bean, lotus

#### Color Words

aqua, beige, black, blue, blush, bronze, brown, coral, crimson, cyan, gold, gray, green, indigo, ivory, jade, khaki, lavender, lilac, lime, magenta, maroon, mauve, mint, navy, olive, orange, peach, pearl, pink, plum, purple, rose, ruby, rust, sable, sand, sapphire, scarlet, silver, teal, violet, white, yellow, sepia

#### Sports Words

rugby, soccer, tennis, golf, boxing, skiing, cycling, diving, hockey, rowing, sailing, skating, sprint, surfing, swimming, racing, bowling, cricket, karate, judo, biking, archery, canoe, curling, fencing, fishing, kayaking, squash, running, walking, jogging, track, chess, lifting, darts, snooker, pool, football, baseball, softball, handball, lacrosse, polo

#### Furniture Words

chair, table, couch, stool, bench, shelf, desk, drawer, sofa, mirror, chest, stand, ottoman, cabinet, dresser, chaise, futon, buffet, armoire, loveseat, rocker, bunkbed, lamp, armchair, recliner, bookcase, settee, vanity, pouffe, hutch, trolley, divan, armrest, curtain, screen, cart, crate, hanger, trunk, hammock

#### Low Similarity List Control Condition Words (Similarity Between 0.0 and 0.3)

oven, player, exam, movie, army, paper, month, disk, road, office, dealer, message, alcohol, hair, ladder, diamond, list, name, mouse, plan, profit, couple, spot, trouble, mark, suit, pace, make, long, mobile, split, kneepad, purse, turbine, fossil, mullet, bonsai, globe

#### High Pair Similarity Control Condition Word Pairs (Pair-Wise Similarity Above 0.7 and Similarity to All Other Words Below 0.3)

“variety range,,’ “town city,” “vehicle truck,” “customer client,” “length height,” “guitar piano,” “garbage trash,” “focus emphasis,” “shop store,” “inside outside,” “anger fear,” “girl woman,” “welcome invite,” “state nation,” “tongue cheek,” “incident accident,” “word phrase,” “female male,” “quiet calm,” “snow rain,” “loan mortgage,” “push pull,” “primary main”

## Footnotes

1 RT $\sim$ set size * block + (set size + block | subject)

## Code Availability

All code has been made publicly available and can be accessed at https://github.com/susanneharidi/memoryscaling.
